# SmCSP4 from aphid saliva stimulates salicylic acid‐mediated defence responses in wheat by interacting with transcription factor TaWKRY76

**DOI:** 10.1111/pbi.14139

**Published:** 2023-08-04

**Authors:** Yong Zhang, Yu Fu, Xiaobei Liu, Frédéric Francis, Jia Fan, Huan Liu, Qian Wang, Yu Sun, Yumeng Zhang, Julian Chen

**Affiliations:** ^1^ State Key Laboratory for Biology of Plant Diseases and Insect Pests, Institute of Plant Protection Chinese Academy of Agricultural Sciences Beijing China; ^2^ PHIM Plant Health Institute Univ Montpellier, INRAE, CIRAD, Institut Agro, IRD Montpellier France; ^3^ Functional and Evolutionary Entomology, Gembloux Agro‐Bio Tech University of Liège Gembloux Belgium; ^4^ Department of Entomology, College of Plant Protection China Agricultural University Beijing China; ^5^ College of Plant Health and Medicine Qingdao Agricultural University Qingdao China

**Keywords:** grain aphid, chemosensory protein, salivary elicitor, salicylic acid, plant immunity, molecular basis

## Abstract

Aphid salivary proteins are critical in modulating plant defence responses. Grain aphid *Sitobion miscanthi* is an important wheat pest worldwide. However, the molecular basis for the regulation of the plant resistance to cereal aphids remains largely unknown. Here, we show that SmCSP4, a chemosensory protein from *S. miscanthi* saliva, is secreted into wheat plants during aphid feeding. Delivery of SmCSP4 into wheat leaves activates salicylic acid (SA)‐mediated plant defence responses and subsequently reduces aphid performance by deterring aphid feeding behaviour. In contrast, silencing *SmCSP4* gene via nanocarrier‐mediated RNAi significantly decreases the ability of aphids to activate SA defence pathway. Protein–protein interaction assays showed that SmCSP4 directly interacts with wheat transcriptional factor TaWRKY76 in plant nucleus. Furthermore, TaWRKY76 directly binds to the promoter of SA degradation gene *Downy Mildew Resistant 6* (*DMR6*) and regulates its gene expression as transcriptional activator. SmCSP4 secreted by aphids reduces the transcriptional activation activity of TaWRKY76 on *DMR6* gene expression, which is proposed to result in increases of SA accumulation and enhanced plant immunity. This study demonstrated that SmCSP4 acts as salivary elicitor that is involved in activating SA signalling defence pathway of wheat by interacting with TaWRKY76, which provide novel insights into aphid‐cereal crops interactions and the molecular mechanism on induced plant immunity.

## Introduction

Plants have evolved complex and accurate defence strategies to prevent or limit damage from herbivorous insects and microbial pathogens (Jones and Dangl, 2006). During pathogen or insect attack, plants respond by producing phytohormones, such as salicylic acid (SA), jasmonic acid (JA) and ethylene (ET), which act as signalling molecules to stimulate downstream defence responses in plants (Berens *et al*., 2017). In general, chewing insects and necrotrophic pathogens mainly activate JA/ET‐mediated defences, whereas hemipterans and biotrophic pathogens primarily induce SA‐mediated defence responses (Züst and Agrawal, 2016).

Plants can rapidly and precisely perceive herbivore cues to activate defences against their attack. Herbivore‐associated molecular patterns (HAMPs) or elicitors in the oral secretions of herbivores play important roles in eliciting plant defences (Acevedo *et al*., 2015). Several HAMPs or elicitors have been identified in the regurgitant and/or saliva of chewing insects including fatty acid conjugates (e.g., volicitin), caeliferins, bruchins and the plant‐derived peptides called inceptins (Alborn *et al*., 1997, 2007; Schmelz *et al*., 2006).

Aphids are important agricultural and forest pests that cause damage by directly drawing phloem sap from sieve tubes and by acting as vectors to transmit various plant viruses (Blackman and Eastop, 2000). During the penetration of plant cells, the stylets of aphids essentially follow an intercellular pathway to reach the sieve elements (Silva‐Sanzana *et al*., 2020; Tjallingii and Esch, 1993). At the early stage of feeding, aphids secrete gelling saliva, which hardens to form a protective sheath around the stylets; subsequently aphids secrete watery saliva containing a number of proteins, which are injected into the plant cells (Cherqui and Tjallingii, 2000; Miles, 1999). To date, many studies have demonstrated that aphid saliva performs important roles in mediating the interactions between aphid and host plant (Elzinga and Jander, 2013; Yates and Michel, 2018). For example, the infiltration of salivary components in the 3–10 kDa fraction obtained from green peach aphid (*Myzus persicae*) induced the expression of defence‐related genes in *Arabidopsis thaliana* and activated resistance against aphids, demonstrating the defence‐eliciting activity of aphid salivary components (De Vos and Jander, 2009). Moreover, green peach aphid *Myzus persicae* salivary proteins Mp10, Mp42, Mp56, Mp57 and Mp58 decreased aphid reproduction when transiently expressed in tobacco plants *Nicotiana benthamiana*, highlighting their potential roles in activating plant defences (Bos *et al*., 2010; Rodriguez *et al*., 2014). Similarly, overexpression of salivary protein Armet secreted by pea aphid *Acyrthosiphon pisum* and infiltration of its purified form in *N. benthamiana* primarily induced SA‐mediated defence responses, which enhanced plant resistance to the bacterial pathogen *Pseudomonas syringae* (Cui *et al*., 2019). *Buchnera aphidicola*‐derived molecular chaperone proteins GroEL and GroES were also identified as elicitors that betray aphids by triggering the plant defence response (Chaudhary *et al*., 2014; Li *et al*., 2022). However, little is known about the mechanisms adopted by host plants to recognize the HAMP‐like molecules or elicitors secreted by aphids.

Like pathogen effectors, salivary effectors have also been identified in aphid saliva, which suppress plant defence responses and contribute to plant susceptibility, thus promoting aphid virulence. The salivary protein C002 of *A. pisum* is the first well‐characterized aphid effector, which is essential for continuous phloem feeding (Mutti *et al*., 2006, 2008). In addition, transient overexpression of genes encoding the MpC002 protein of *M. persicae* and the Me10 and Me23 proteins of potato aphid *Macrosiphum euphorbiae* in *N. benthamiana* resulted in a significant increase in aphid fecundity, implying that these effectors suppress plant defence (Atamian *et al*., 2013; Bos *et al*., 2010; Elzinga *et al*., 2014; Kettles and Kaloshian, 2016). Ectopic expression of bird cherry‐oat aphid *Rhopalosiphum padi* genes encoding candidate effectors RpC002 and Rp1 in barley *Hordeum vulgare* enhanced susceptibility to aphids by inhibiting the expression of defence‐related genes (Escudero‐Martinez *et al*., 2020).

The chemosensory proteins (CSPs) of insects mainly function as carriers of pheromones and odorants and are typically expressed in diverse chemosensory organs, including antennae, mouthparts, and other chemosensory structures (Pelosi *et al*., 2018). CSPs have been identified in various body parts of insects, including the thorax, legs and reproductive organs, and play a role in insect development, mating, leg regeneration, immune responses, pesticide resistance, and mediating interactions with the defence‐related molecules of the host plant (Peng *et al*., 2021; Stathopoulos *et al*., 2002). CSPs have also been identified in the salivary glands and watery saliva of several hemipteran insects, such as *M. persicae*, *R. padi*, black cherry aphid *Myzus cerasi* and brown planthopper *Nilaparvata lugens* (Ji *et al*., 2013; Thorpe *et al*., 2016). When transiently expressed in *N. benthamiana*, the gene encoding Mp10, a CSP detected in *M. persicae* saliva, inhibited flg22‐induced defence responses but induced chlorosis and local cell death, resulting in a significant reduction in aphid fecundity (Bos *et al*., 2010; Rodriguez *et al*., 2014).

Grain aphid *Sitobion miscanthi*, which was wrongly designated previously as English grain aphid *Sitobion avenae* in China, is one of the major insect pests of cereal crops worldwide and the most dominant aphid species in the major wheat *Triticum aestivum*‐growing regions of China (Jiang *et al*., 2019; Zhang, 1999). *S. miscanthi* can cause huge yield losses by directly drawing phloem sap and transmitting various plant viruses, such as barley yellow dwarf virus (Blackman and Eastop, 2000). *S. miscanthi* infestation mainly activates the SA‐associated defence signalling pathway in wheat (Huang *et al*., 2019), and the watery saliva secreted by *S. miscanthi* induces the expression levels of several SA‐related genes, thus enhancing resistance against aphids (Zhang *et al*., 2017a). Four *CSP* genes have been identified in *S. miscanthi* based on the transcriptome profiling of its salivary glands (Zhang *et al*., 2017b). However, the roles and molecular mechanisms of SmCSPs in modulating wheat defence responses remain unknown. Here we identified a secreted CSP of *S. miscanthi*, SmCSP4, which can effectively activate SA‐mediated plant defence response and enhance wheat resistance against aphids. We demonstrate that SmCSP4 interacts with the TaWRKY76 transcription factor of wheat in the plant cell nucleus and suppresses the SA degradation pathway by inhibiting the binding of TaWRKY76 to the *downy mildew resistant 6* (*DMR6*) gene promoter.

## Results

### Identification and characterization of *SmCSP4*

**Figure 1:**
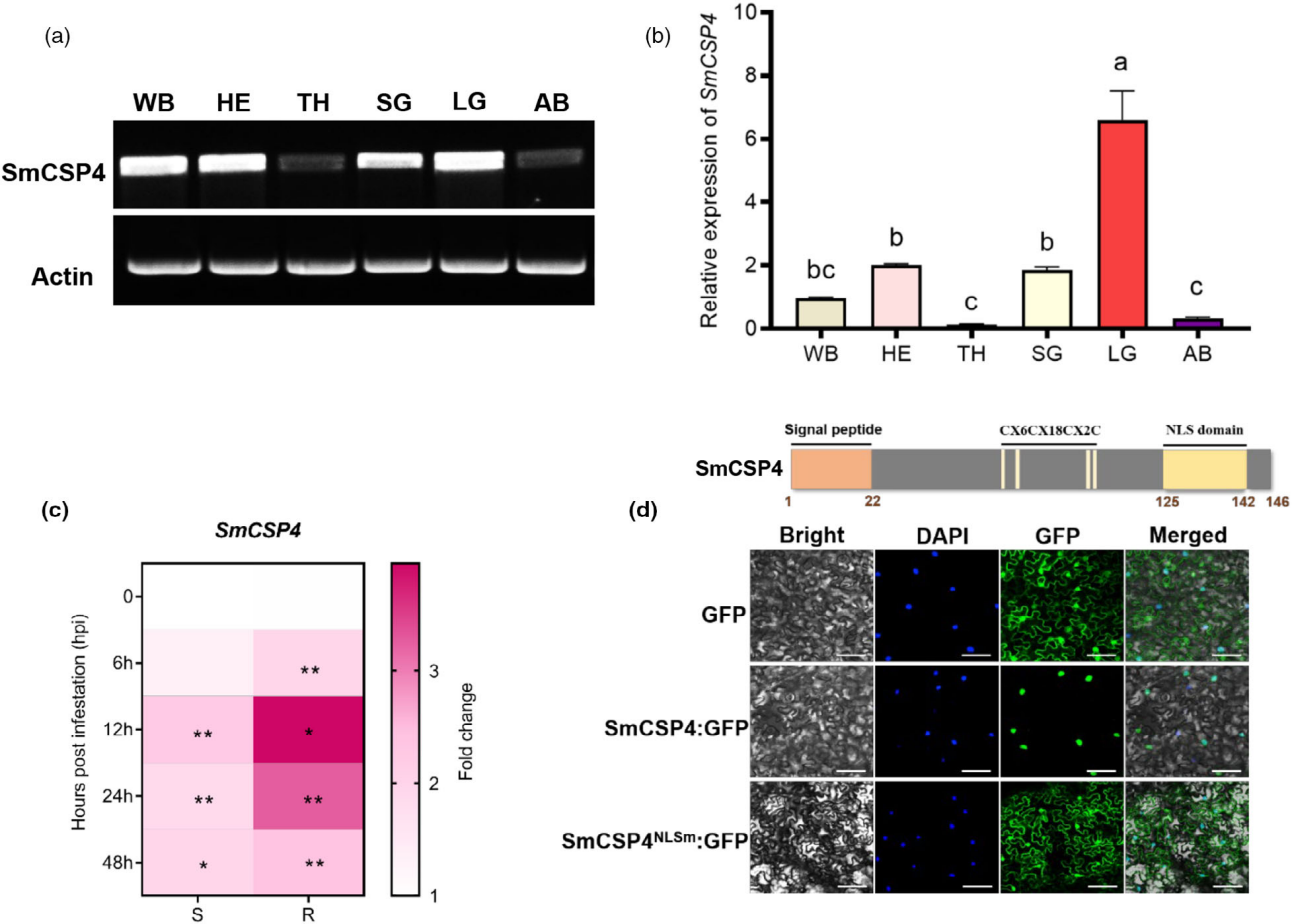
Characterization of SmCSP4 from *S. miscanthi*. (a) Detection of SmCSP4 in various aphid tissues using RT‐PCR. (b) Detection of *SmCSP4* in different aphid tissues by RT‐qPCR. *β‐actin* and *NADH hydrogenase* were used as internal reference genes. Standard error (SE) is represented by the error bar. Different lower‐case letters above the bars indicate significant differences among treatments (*P* < 0.05, one‐way ANOVA followed by Duncan's multiple range test). Abbreviation for tissues: Whole body of apterous adults (WB), heads (HE), antennae (AN), thorax (TH), abdomen (AB), salivary glands (SG), and legs (LG). (c) The expression levels of *SmCSP4* in *S. miscanthi* after feeding on aphid‐susceptible wheat variety (S) Mingxian169 and aphid‐resistant (R) wheat variety Zhong4wumang at different time points. *β‐actin* and *NADH dehydrogenase* were used as internal reference genes. Three replicates were conducted for each treatment. Asterisks above bars indicate significant differences between controls and treatments (*P < 0.05; **P < 0.01; Student's t test). (d) Subcellular localization of SmCSP4 or SmCSP4^NLSm^ in *N. benthamiana*. GFP was used as control groups. The images were taken 24 h after agroinfiltration using confocal laser scanning microscopy. Bar = 25 μm.

*SmCSP4* is 441 bp in length and is predicted to encode a 146‐amino acid protein, with a molecular weight of 16.54 kDa and a signal peptide (SP) at its N terminus. Phylogenetic analysis revealed that SmCSP4 is the most closely related to the *A. pisum* CSP4 (ApisCPS4) protein (Figure S1A). Sequence alignment showed that SmCSP4 contains four conserved cysteine residues (CX_6_CX_18_CX_2_C) and exhibits high similarity to the homologues from *A. pisum*, *M. persicae*, and *A. gossypii* (Figure S1B). The results of RT‐PCR and RT‐qPCR indicated that *SmCSP4* was expressed in different tissues of aphids, including the whole body, head, salivary gland, leg, abdomen and thorax, and its expression was significantly higher in legs compared with the other tissues (Figure 1a,b). The transcript level of *SmCSP4* in aphids significantly increased after feeding on wheat plants, but the gene exhibited a higher fold change after feeding on the aphid‐resistant than on the aphid‐susceptible wheat variety (Figure 1c).

A nuclear localization signal (NLS) sequence from 126 to 142 amino acids was predicted at the C‐terminal end of SmCSP4 (Figure 1d). To further confirm the subcellular localization of SmCSP4, the *SmCSP4‐GFP* fusion, Sm*CSP4* ^*NLSm*^ *‐GFP* with mutant NLS fusion or *GFP* alone (control) was transiently expressed in *N. benthamiana* leaves. In the control group expressing only *GFP* under the control of the cauliflower mosaic virus (CaMV) *35S* promoter, fluorescence was detected throughout the cytoplasm and nucleus. However, in leaves transformed with the *SmCSP4‐GFP* fusion, fluorescence was observed only in the nucleus, indicating that SmCSP4 localizes to the nucleus. However, fluorescence was observed in both cytoplasm and nucleus of leaves expressing Sm*CSP4* ^*NLSm*^ *‐GFP* (Figure 1d).

### Aphids secrete SmCSP4 into the plant cell during feeding

**Figure 2:**
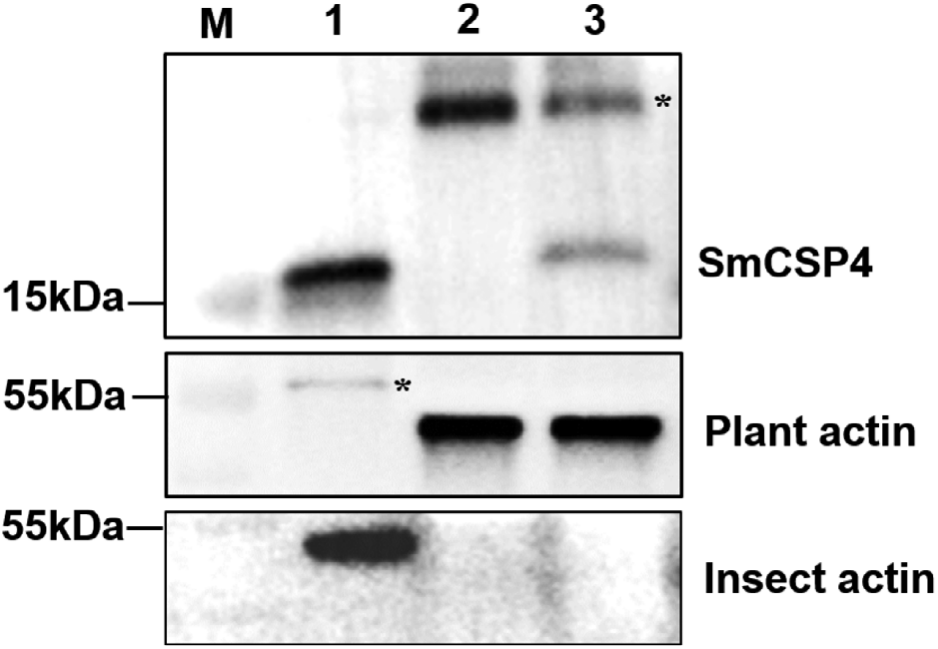
SmCSP4 is a secreted salivary protein. Western blot analysis of SmCSP4. Lane 1, protein extracted from aphid whole body; lane 2, protein extracted from wheat leaves without aphid feeding; lane 3, protein extracted from wheat leaves fed upon by aphids. Anti‐actin antibody of plants or insects was used to detect the loading of lanes 1–3. M: Protein marker. The asterisk denotes an unspecific band.

SmCSP4 is believed to be a component of aphid watery saliva. Western blotting was performed to verify that the SmCSP4 was secreted into plant cells. As shown in Figure 2, the results revealed that SmCSP4 was present in the whole body of *S. miscanthi* and in aphid‐infested wheat leaves, but was not detected in wheat leaves without aphid infestation, these results suggested that SmCSP4 is secreted by aphids into wheat plants during feeding.

### SmCSP4 induces SA‐mediated plant immunity

**Figure 3:**
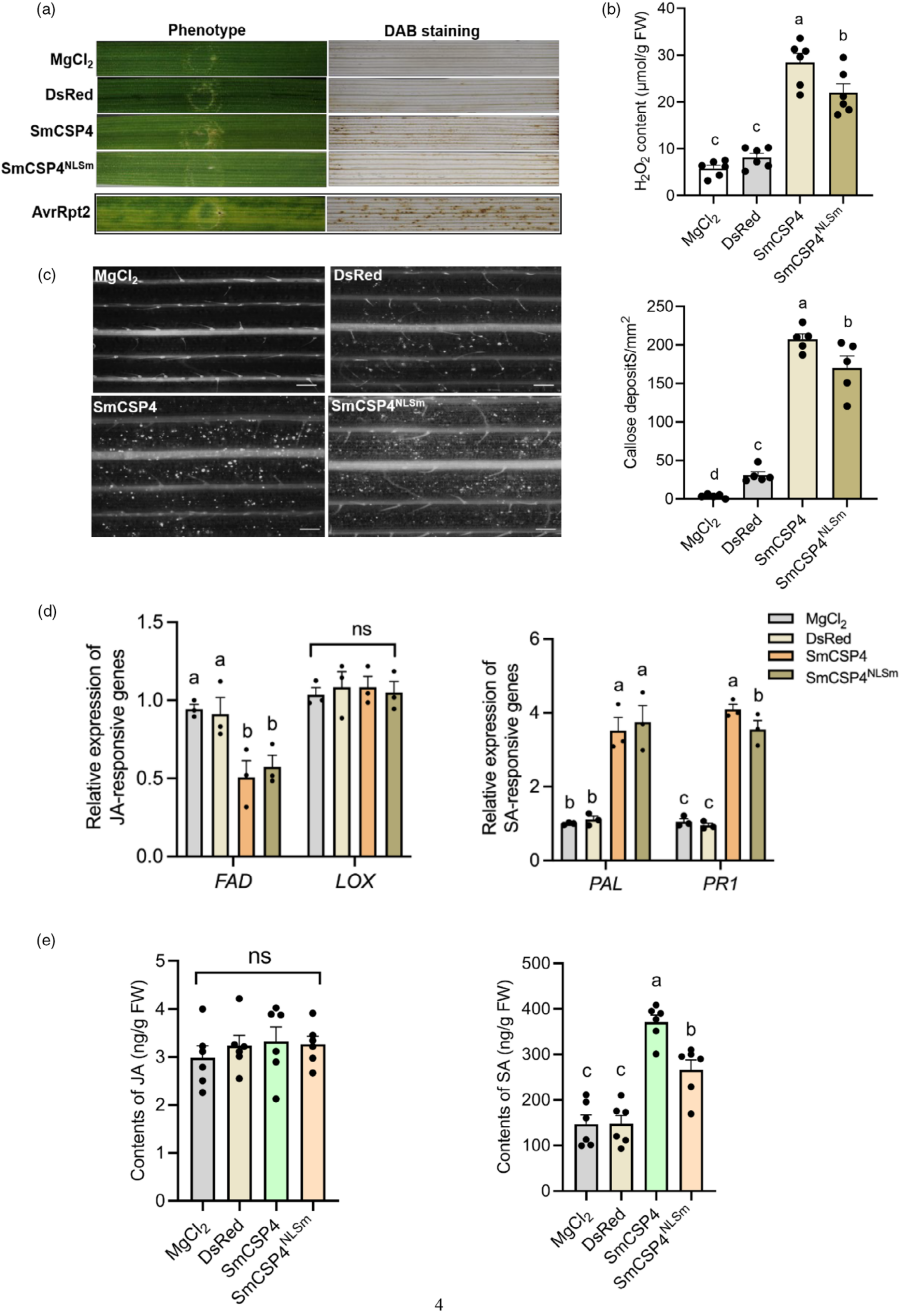
SmCSP4 activated salicylic acid‐mediated signalling defence pathway in plant. (a) Phenotype and H_2_O_2_ accumulation in wheat leaves (*var*. Zhong4wumang) after infiltration with *P. fluorescens* EtAnH carrying pEDV6: *SmCSP4* or pEDV6: *SmCSP4* ^*NLSm*^ at 2 days. Leaves infiltrated with MgCl_2_, pEDV6:*DsRed* or pEDV6:*AvrRpt2* were set as blank, negative and positive controls, respectively. (b) Content of H_2_O_2_ in wheat leaves expressed with *SmCSP4* or *SmCSP4* ^*NLSm*^ after 2 days of infiltration (*n* = 6). Different lower‐case letters above the bars indicate significant differences between controls and treatments (*P* < 0.05; one‐way ANOVA followed by Duncan's multiple range test). (c) Aniline blue staining was performed to examine callose deposition in the wheat leaves infiltrated with EtAnH carrying pEDV6: *SmCSP4* or pEDV6:*SmCSP4* ^*NLSm*^ at 2 days using epifluorescence microscopy. Leaves treated with MgCl_2_ and pEDV6:*DsRed* were set as controls. Bar = 330 μm. Five replicates were used for analysis. (d) The expression levels of JA synthesis‐related genes *FAD* and *LOX*, and SA signalling pathway‐associated genes *PAL* and *PR1* in wheat leaves infiltrated with MgCl_2_ solution or *P. fluorescens* EtAnH carrying pEDV6:*DsRed*, pEDV6:*SmCSP4* or pEDV6:*SmCSP4* ^*NLSm*^ at 2 days (*n* = 3). (e) Endogenous JA and SA contents in wheat leaves inoculated with MgCl_2_ solution or *P. fluorescens* EtAnH carrying pEDV6:*DsRed*, pEDV6:*SmCSP4* or pEDV6:*SmCSP4* ^*NLSm*^. Six replicates were conducted for each treatment. Data in the bar chart are represented as mean ± SE.

Wheat is the major host plants of *S. miscanthi*. To directly determine whether SmCSP4 of *S. miscanthi* is capable of modulating wheat plant defence, the SmCSP4 or SmCSP4^NLSm^ was delivered into wheat leaves using the *P. fluorescens* EtAnH‐mediated delivery system. The infiltration of EtAnH expressing SmCSP4 induced slight chlorotic symptoms and resulted in a significant H_2_O_2_ accumulation in the infiltrated region of leaves, and also, the H_2_O_2_ content in SmCSP4‐treated leaves was significantly higher than that in SmCSP4^NLSm^‐infiltrated groups (Figure 3a,b). In addition, significantly more callose deposits were detected in wheat leaves expressing SmCSP4 or SmCSP4^NLSm^ when compared to controls, but the number of callose deposits in SmCSP4^NLSm^‐treated leaves was lower than that in leaves delivered with SmCSP4 (Figure 3c).

Gene expression analysis showed that the JA‐responsive gene *FAD* was significantly down‐regulated in SmCSP4 or SmCSP4^NLSm^‐delivered wheat leaves at 2 dpi, and the expression levels of *LOX* had no significant changes when compared to control. However, the SA biosynthesis gene *PAL* and SA pathway marker gene *PR1* were all significantly up‐regulated in wheat leaves delivering with SmCSP4 or SmCSP4^NLSm^ (Figure 3d). In addition, the delivery of SmCSP4 or SmCSP4^NLSm^ into wheat leaves significantly increased the SA levels at 2 dpi compared with the control, but no significant change was observed in the JA levels. Moreover, the expression levels of *PR1* and SA levels in SmCSP4‐treated leaves were significantly higher than those in SmCSP4^NLSm^‐treated leaves (Figure 3e). The delivery of SmCSP4 and SmCSP4^NLSm^ into wheat leaves was confirmed using Western blot (Figure S2). These results suggest that SmCSP4 is involved in the activation of SA‐associated defence pathways in wheat plants, and NLS domain of SmCSP4 is important for the induction of wheat SA‐mediated plant defence.

### *SmCSP4* expression in wheat enhances plant resistance against *S. miscanthi*

**Figure 4:**
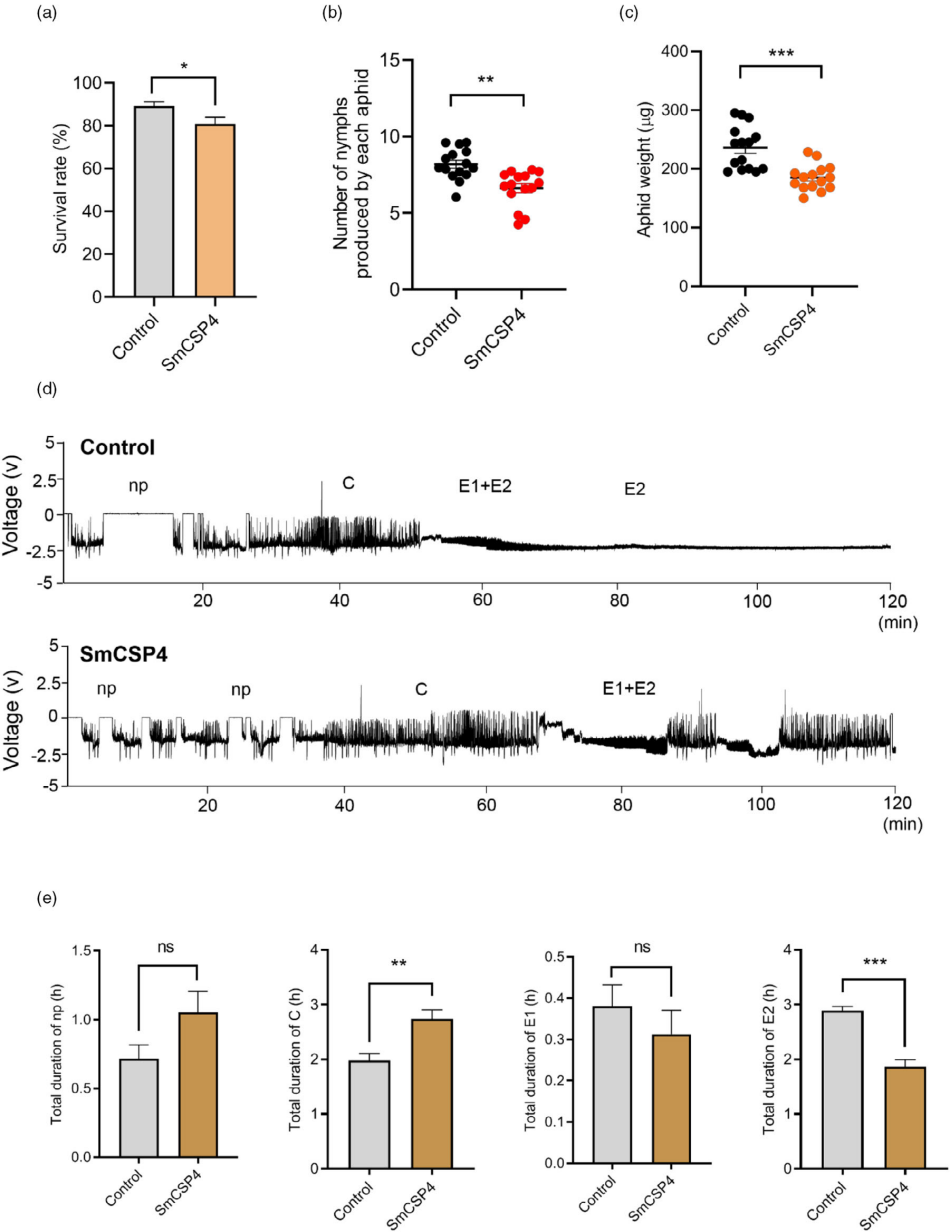
Expression of SmCSP4 in wheat‐reduced aphid performance. (a) The survival rate of *S. miscanthi* when feeding on wheat leaves (*var*. Zhong4wumang) delivered with SmCSP4. Fifteen biological replicates were performed for each group. (b) Number of nymphs produced by *S. miscanthi* after feeding on wheat leaves infiltrated with pEDV6:*DsRed* (control) or pEDV6: *SmCSP4* for 5 days. Fifteen biological replicates were performed for each group. (c) Body weight of *S. miscanthi* after feeding on wheat leaves inoculated with pEDV6:*DsRed* (control) or pEDV6:*SmCSP4* for 7 days. Fifteen biological replicates were performed for each group. Asterisks above the bars indicate significant differences between controls and treatments (*P < 0.05; **P < 0.01; ***P < 0.001; Student's *t* test). (d) Representative EPG waveforms of *S. miscanthi* feeding on wheat leaves delivered with DsRed (control) and SmCSP4, respectively. (e) Feeding behaviour parameters of *S. miscanthi*, including durations of nonprobing (np), stylet probing (C), salivation (E1) and phloem ingestion (E2) when feeding on wheat leaves treated with pEDV6: *DsRed* (control) or pEDV6: *SmCSP4*. Ten biological replicates were performed for each treatment. Asterisks above the bars indicate significant differences between controls and treatments (*P < 0.05; **P < 0.01; ***P < 0.001; Mann–Whitney *U* test.). All data are represented as mean ± SE.

The survival rate of aphids on *SmCSP4*‐infiltrated wheat leaves was significantly lower than that on control leaves (Figure 4a). In addition, the fecundity and weight of aphids feeding on *SmCSP4*‐infiltrated wheat leaves were significantly decreased compared with the control groups (Figure 4b,c).

Moreover, the feeding behaviour of aphids on wheat leaves infiltrated with *SmCSP4* was negatively affected (Figure 4d,e). The duration of phloem ingestion (E2) was significantly lower in wheat leaves infiltrated with *SmCSP4* than in leaves infiltrated with the DsRed (control). Contrarily, the durations of stylet probing activity (C) waveforms in SmCSP4‐expressed leaf areas were significantly higher than those in the control groups. These results indicate that SmCSP4 reduces aphid performance and enhances host resistance against aphids.

We also found that the SA content showed a significant increase in wheat leaves infested with *S. miscanthi*; however, this increase in SA content was greater in the aphid‐resistant than in the aphid‐susceptible wheat variety (Figure S3A). Exogenous SA application up‐regulated the transcript levels of SA‐responsive genes (*PAL*, *PR1* and *PR5*) and induced significant callose deposition in wheat leaves (Figure S3B,C). No significant differences were observed in the survival rates of aphids feeding on SA‐treated wheat leaves compared to the control, but the number of nymphs produced by aphids was significantly reduced after feeding on SA‐treated plants (Figure S3D). These results suggest that the SmCSP4‐induced increase in SA levels enhanced wheat resistance against aphids.

### CSP4 orthologs from other aphid species enhance SA levels and plant resistance

**Figure 5:**
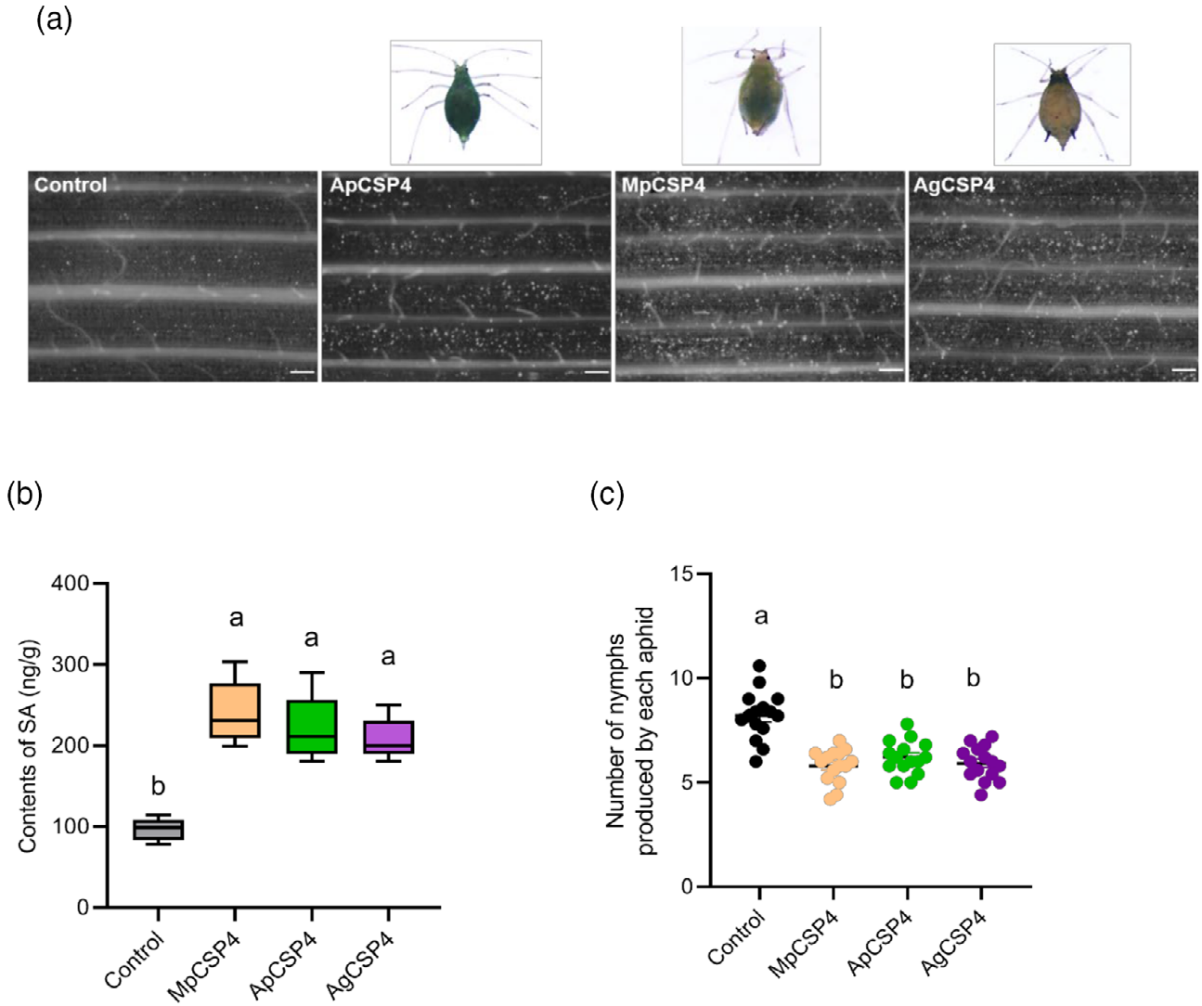
SmCSP4 orthologs from *M. persicae*, *A. pisum* and *A. gosspyii* enhanced SA levels and wheat resistance. (a) Callose deposition in wheat leaves (*var*. Zhong4wumang) expressed with CSP4 orthologs from *A. pisum* (ApCSP4), *M. persicae* (MpCSP4) and *A. gosspyii* (AgCSP4) at 2 days using aniline blue staining. Bar = 330 μm. (b) Endogenous SA contents in wheat leaves inoculated with *P. fluorescens* EtAnH carrying pEDV6: *MpCSP4*, pEDV6: *ApCSP4* or pEDV6: *AgCSP4* respectively. Five replicates were conducted for each treatment. Leaves infiltrated with pEDV6: *DsRed* was used as control. (c) Number of nymphs produced by *S. miscanthi* after feeding on wheat leaves delivered with DsRed, ApCSP4, MpCSP4 or AgCSP4, respectively, for 5 days. Fifteen biological replicates were performed for each treatment. Standard error of the mean (SE) is represented by the error bar. Different lower‐case letters above the bars indicate significant differences between controls and treatments (*P* < 0.05, one‐way ANOVA followed by Duncan's multiple range test).

More callose deposits were observed in wheat leaves expressing CSP4 orthologs from *M. persicae* (MpCSP4), *A. pisum* (ApCSP4) and cotton aphid (*Aphis gossypii*) (AgCSP4) (Figure 5a). In addition, the SA content significantly increased in wheat leaves delivering with MpCSP4, ApCSP4 and AgCSP4 at 2 dpi (Figure 5b). Moreover, aphid fecundity significantly decreased after feeding on wheat leaves expressing these three CSP4 compared with the controls (Figure 5c). This suggests that aphid CSP4 orthologs possess conserved functions that elicit plant defence.

### Silencing of *SmCSP4 via* nanocarrier‐mediated RNAi activates weaker SA‐associated defence responses but reduces aphid fecundity

**Figure 6:**
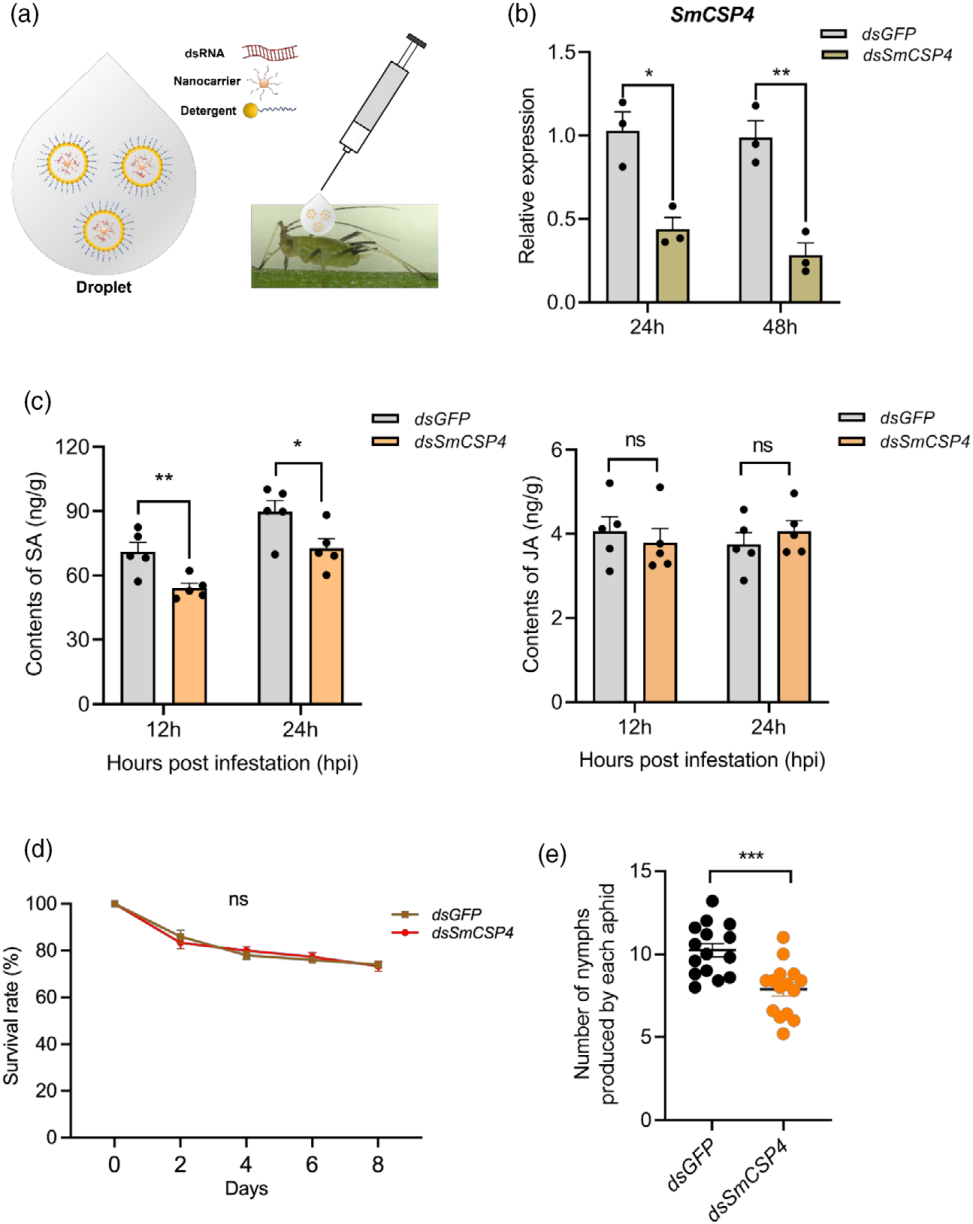
*SmCSP4*‐silenced aphid via nanocarrier‐mediated RNAi activated weaker defence responses. (a) Schematic diagram for applying the nanocarrier‐mediated transdermal dsRNA delivery system. The dsRNA/nanocarrier/detergent droplet was dropped on the notum of *S. miscanthi* using microinjector. (b) Relative expression levels of *SmCSP4* at 24 and 48 h after *dsSmCSP4*/nanocarrier/detergent or *dsGFP/*nanocarrier/detergent (control) treatments. Three biological replicates were conducted for each group. (c) Content of SA and JA in wheat leaves after infestation of aphids treated with ds*GFP* or ds*SmCSP4* at 12 and 24 dpi. Five replicates were performed for each treatment. (d) Survival rate of *SmCSP4*‐silenced aphids after feeding on aphid‐resistant wheat variety Zhong4wumang at four different time points. Fifteen biological replicates were conducted (*n* = 15). (e) Number of nymphs produce by each aphid treated with ds*SmCSP4* or ds*GFP* after feeding on wheat plants (*var*. Zhong4wumang) for 5 days. Fifteen biological replicates were conducted. All data shown are mean ± SE. ‘ns’ indicates no significant differences between controls and treatments. Asterisks above bars and lines indicate significant differences between controls and treatments (*P < 0.05; **P < 0.01; ***P < 0.001; Student's *t* test).

A schematic representation of nanocarrier star polycation (SPc)‐based transdermal dsRNA delivery is presented in Figure 6a. The transcript level of *SmCSP4* was significantly reduced in *S. miscanthi* treated with 500 ng/μL dsRNA of *SmCSP4* for 24 h. After 48 h of dsRNA treatment, the expression level of *SmCSP4* further decreased to 0.28‐fold, which was significantly lower than the transcript levels observed in the control (Figure 6b). *SmCSP4*‐silenced aphids induced less SA production in wheat leaves compared with the control groups; however, infestation by *SmCSP4*‐silenced aphids had no significant effects on the JA content of wheat leaves (Figure 6c).

Effects of *SmCSP4* silencing on aphid survival, fecundity and olfactory behaviour were also investigated. As presented in Figure 6d,e, *SmCSP4* knockdown had no significant effects on *S. miscanthi* survival rate but significantly reduced their fecundity compared with the control (*dsGFP*). Furthermore, the olfactory behaviour assay results indicated SmCSP4 is not involved in the recognition of chemical cues that the repellent effect of aphid alarm pheromone (*E*)‐β‐farnesene (EBF) and attractive activity of wheat plant volatiles on *S. miscanthi* was not affected by *SmCSP4* knockdown (Figure S4). These results suggested that SmCSP4 is essential for aphid fitness but also involved in the induction of SA defence in wheat as elicitor.

### SmCSP4 interacts with TaWRKY76 in plant cell nucleus

**Figure 7:**
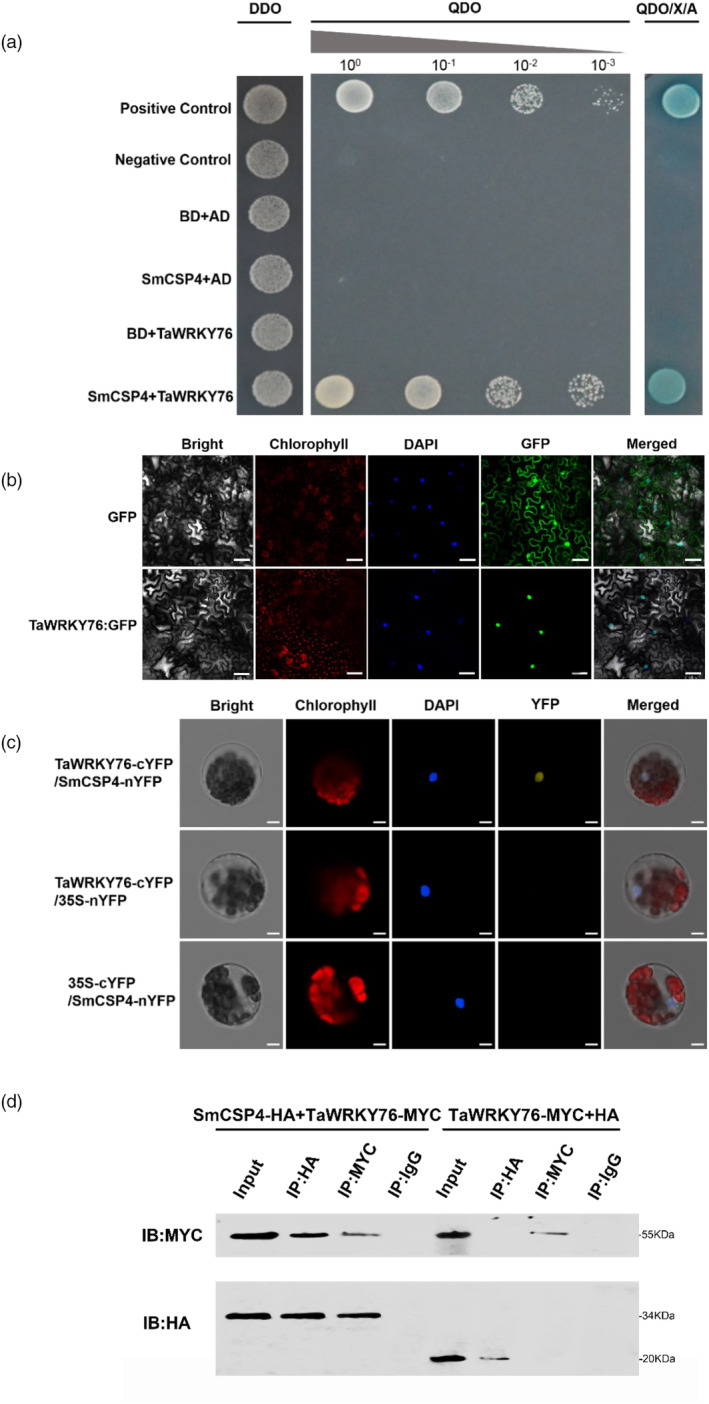
SmCSP4 interacted with TaWRKY76 of wheat. (a) Yeast two‐hybrid assays to detect the interaction between SmCSP4 (Bait) and TaWRKY76 (Prey). Gradient concentrations of the yeast cells co‐transformed with SmCSP4 and TaWRKY76 were assayed for growth on SD‐Trp‐Leu‐His‐Ade LB plates containing X‐α‐gal and Aureobasidin A (QDO/X/A). Yeast cells co‐transformed with pGBKT7‐53+pGADT7‐T and pGBKT7‐Lam+pGADT7‐T are used as positive and negative controls, respectively. (b) Subcellular localization of TaWRKY76 in *N. benthamiana*. The images were taken 2 days after agroinfiltration using confocal microscopy. Bar = 20 μm. (c) BiFC assays for the interaction of SmCSP4 with TaWRKY76 in *N. benthamiana* protoplasts were introduced with a mixture of *SmCSP4*‐*nYFP* and *TaWRKY76*‐*cYFP* constructs. YFP signals were observed at 20 h post‐incubation using laser scanning confocal microscope. Infiltration with *Agrobacterium* expressing the SmCSP4 or TaWRKY76 alone was used as controls. Scale bar = 20 μm. (d) Confirmation of the interaction between SmCSP4 and TaWRKY76 using Co‐IP assays. Western blots of total proteins from *N. benthamiana* leaves transiently expressing the labelled proteins eluted from anti‐HA or anti‐MYC magnetic beads were detected with the anti‐MYC or anti‐HA antibody. IP with IgG antibody served as negative control. The sizes of SmCSP4: HA and TaWRKY76: MYC bands were 34 and 55 kDa, respectively.

To identify the SmCSP4‐interacting proteins, a Yeast two‐hybrid (Y2H) assay was conducted using SmCSP4 as the bait to screen an aphid‐infested wheat cDNA library. Ten CDS full sequences of putative positive targets of SmCSP4 were obtained preliminary. Sequence analysis revealed that all these identified candidate SmCSP4‐interacting proteins were 26S proteasome non‐ATPase regulatory subunit 14 homologue (GenBank accession no. XM_044500535) and transcription factor TaWRKY76 (GenBank accession no. XM_044541645). As SmCSP4 localizes to the nucleus, we focused on the transcriptional factor of wheat TaWRKY76. Phylogenetic analysis of the predicted amino acid sequences of Arabidopsis WRKY proteins revealed that TaWRKY76 clustered into group IIa of the WRKY superfamily (Figure S5). Full‐length *TaWRKY76* CDS was used to generate the prey construct to confirm the interaction between TaWRKY76 and SmCSP4 *via* Y2H assays (Figure 7a). Subcellular localization of TaWRKY76 was examined by transiently expressing the *TaWRKY76:GFP* fusion in *N. benthamiana* leaves. The fluorescence signal of TaWRKY76‐GFP was observed only in the nucleus of *N. benthamiana* cells (Figure 7b), indicating that TaWRKY76 localizes to the nucleus.

The SmCSP4‐TaWRKY76 interaction in plant cells was verified by performing bimolecular fluorescence complementation (BiFC) assays. *SmCSP4‐nYFP* and *TaWRKY76‐cYFP* were co‐expressed in *N. benthamiana* protoplasts. YFP signal was observed in the nucleus of *N. benthamiana* protoplasts but not in the cytoplasm (Figure 7c), indicating that SmCSP4‐nYFP and TaWRKY76‐cYFP are localized to and interact in the nucleus of plant cells. We further verified the interaction between SmCSP4 and TaWRKY76 *via in vivo* co‐immunoprecipitation (Co‐IP) assays in *N. benthamiana* leaves agroinfiltrated with SmCSP4‐HA and TaWRKY76‐MYC or with HA and TaWRKY76‐MYC (control). As presented in Figure 7d, in leaves co‐infiltrated with SmCSP4‐HA and TaWRKY76‐MYC, the SmCSP4‐HA and TaWRKY76‐MYC recombinant proteins could be immunoprecipitated by anti‐HA and anti‐MYC agarose beads, respectively; however, no target protein was immunoprecipitated in the control groups. These results further confirmed the interaction between SmCSP4 and TaWRKY76 *in vivo*.

### Silencing of *TaWRKY76* induces the SA defence response pathway in wheat and reduces aphid performance

**Figure 8:**
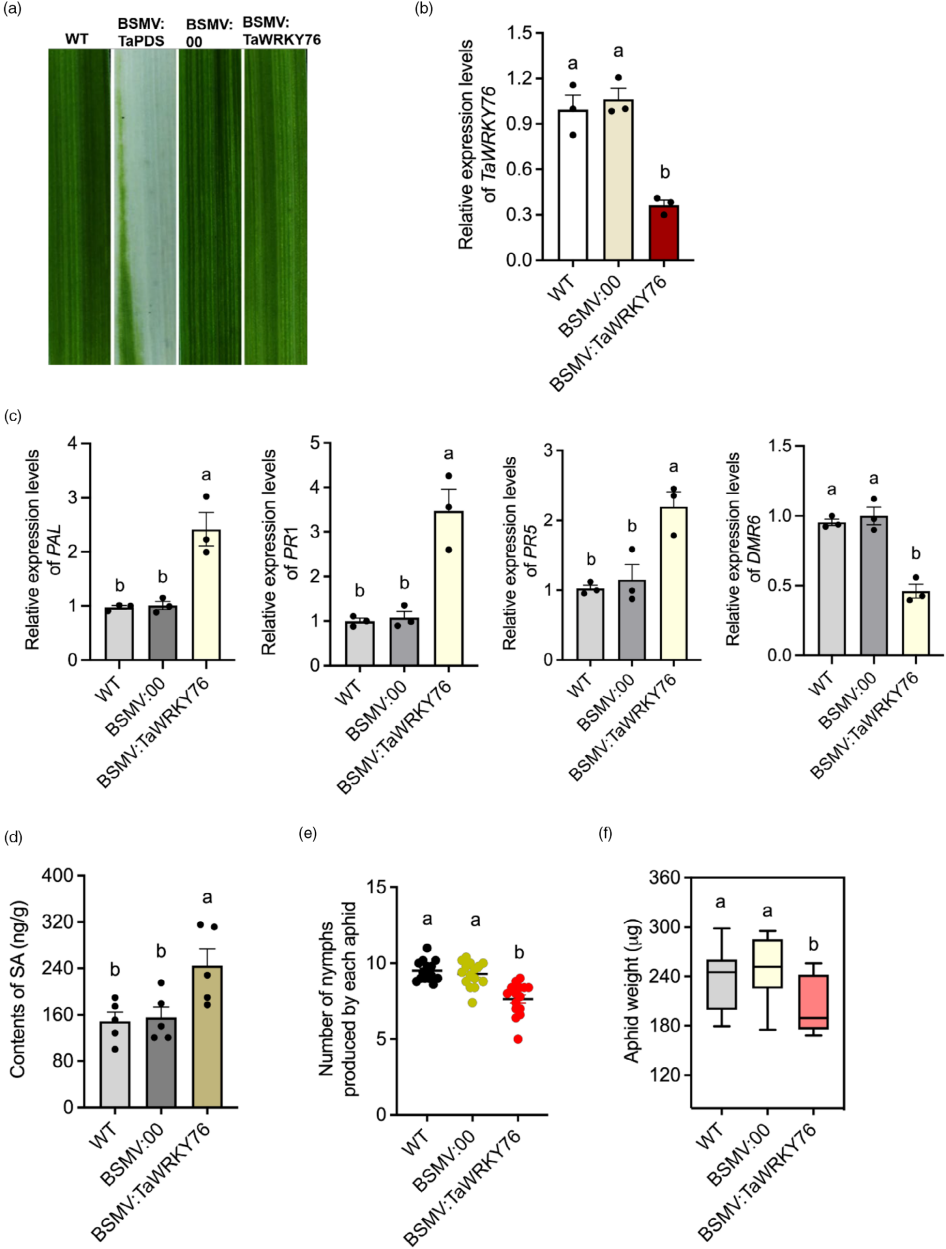
Silencing of *TaWRKY76* activated salicylic acid‐mediated wheat defence responses. (a) The second leaves wheat cultivar Mingxian169 were inoculated with barley stripe mosaic virus BSMV:00 (Control) and recombinant BSMV:*TaPDS*, BSMV:*TaWRKY76* at two‐leaf stage, and the phenotypes were observed and photographed 10 days after inoculation. (b) Expression levels of *TaWRKY76* in wheat leaves inoculated with BSMV:*TaWRKY76* at 5 days (*n* = 3). Wide type (WT) and wheat leaves inoculated with BSMV:00 were set as control groups. (c) Expression levels of SA signalling pathway associated genes *PAL*, *PR1*, *PR5* and *DMR6* after silencing of *TaWRKY76* in wheat leaves. *β‐Actin* was set as internal reference gene. Three replicates were performed for each treatment. (d) Content of SA in wide type (WT) and wheat leaves inoculated with BSMV:00 or BSMV:*TaWRKY76* at 5 days (*n* = 5). (e) Number of nymphs produced by each aphid in 5 days after feeding on *TaWRKY76*‐silenced wheat leaves (*n* = 15). (f) Aphid weight at 7 days post‐feeding on *TaWRKY76*‐silenced wheat leaves (*n* = 15). WT and wheat leaves inoculated with BSMV:00 were set as control groups. All data shown are mean ± SE. Different letters above the bars indicate significant differences among treatments (*P* < 0.05, One‐way ANOVA followed by Duncan's multiple range test).

To determine the function of TaWRKY76 in modulating SA‐associated plant defence, the transcription of *TaWRKY76* in the aphid‐susceptible wheat cultivar Mingxian169 was down‐regulated by virus‐induced gene silencing (VIGS) using the BSMV vector. Wheat leaves infiltrated with the BSMV:*TaPDS* recombinant vector exhibited severe photobleaching at 10 dpi but no phenotypic change, suggesting that the VIGS system effectively worked in this study (Figure 8a). Gene expression analysis revealed that the transcript level of *TaWRKY76* was significantly reduced at 24 hpi and further decreased to 0.36‐fold at 48 hpi (Figure 8b).

SA‐responsive genes, including *PAL*, *PR1* and *PR5* were significantly up‐regulated (Figure 8c), but the expression levels of *DMR6*, an enzyme involved in SA hydroxylation, the main pathway that mediates SA degradation in plants (Van Damme *et al*., 2008; Zhang *et al*., 2017), was significantly decreased in *TaWRKY76* knockdown plants. And the SA content of *TaWRKY76*‐silenced wheat leaves was significantly increased compared with the control (Figure 8d). In addition, the fecundity and weight of aphids were significantly reduced after being fed on wheat leaves inoculated with BSMV:*TaWRKY76* compared with wild‐type plants and wheat leaves inoculated with BSMV:*00* (Figure 8e,f), indicating that TaWRKY76 is involved in the suppression of SA‐mediated wheat defence responses against *S. miscanthi*.

### SmCSP4 suppresses the transcriptional activation activity of TaWRKY76 on *DMR6* expression

**Figure 9:**
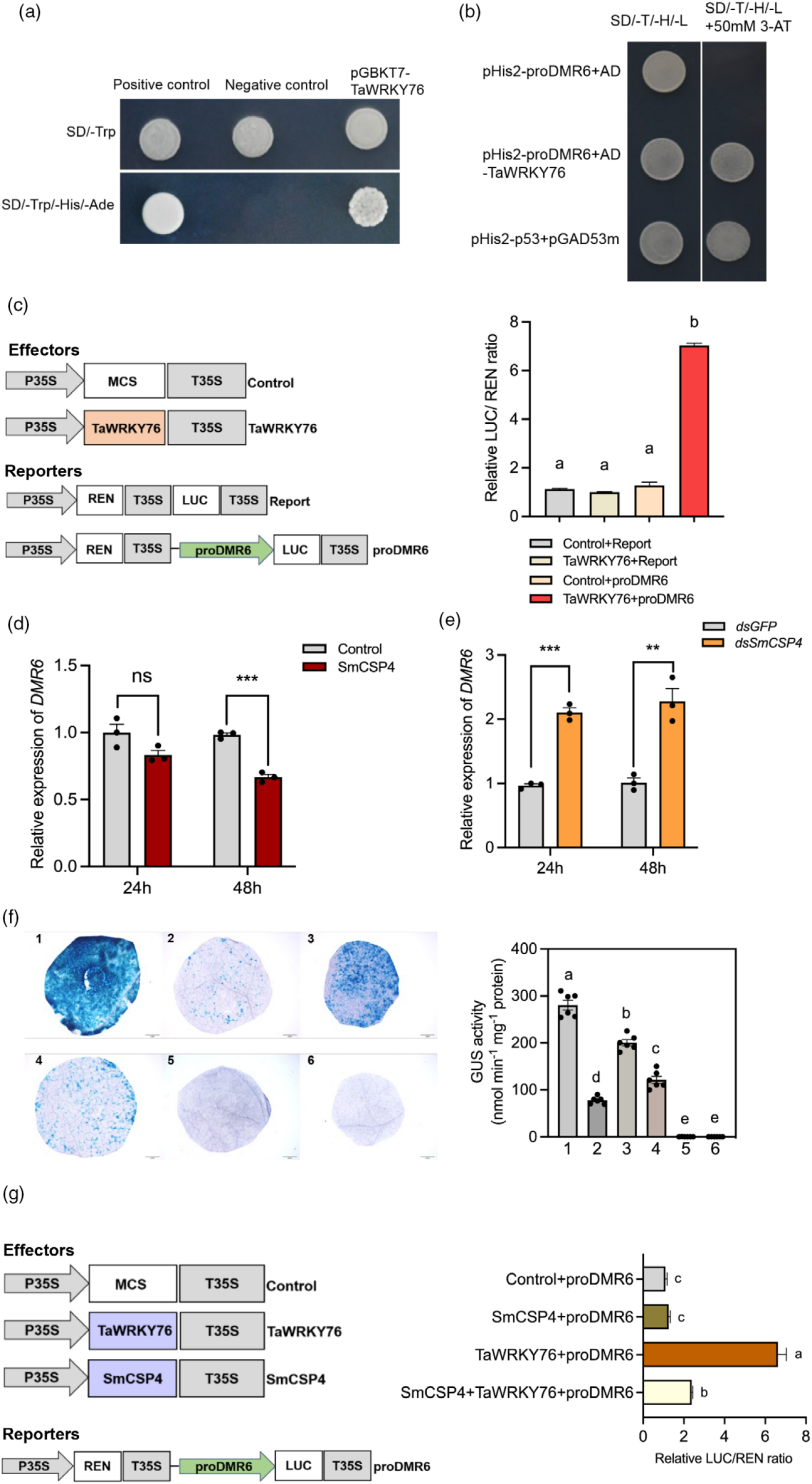
SmCSP4 inhibits the transcriptional activation activity of TaWRKY76 on *DMR6* expression. (a) Yeast assays showing the transcriptional activation activity of TaWRKY76. Yeast cells co‐transformed with pGBKT7‐53+pGADT7‐T and pGBKT7‐Lam+pGADT7‐T are used as positive and negative controls, respectively. (b) Yeast one‐hybrid assay (Y1H) examined the interaction of the TaWRKY76 with *DMR6* promoter. AD‐*TaWRKY76* and pHIS2‐*proDMR6* constructs were co‐transformed into yeast Y187, and recombined cells were grown on SD/‐Trp/‐His or SD/‐Trp/‐Leu/‐His containing 50 mm 3‐AT. Yeast cells co‐transformed with pGBKT7‐*p53* and pGAD53m are used as positive control. (c) Dual‐luciferase reporter assays detected the binding activity of TaWRKY76 in the promoter of *DMR6*. pGreenII0800‐*proDMR6*‐LUC and pGreenII62‐*TaWRKY76*‐SK were transiently transformed into *N. benthamiana* leaves to detect the luminescence. The relative luciferase activities in different samples were calculated by normalizing the LUC values against REN (*n* = 6). All data presented in the boxplots were shown as mean ± SE. Different letters above the bars indicate significant differences among treatments (*P* < 0.05, One‐way ANOVA followed by Duncan's multiple range test). (d) The expression levels of *DMR6* on wheat leaves infiltrated with pEDV6:*SmCSP4* or pEDV6:*DsRed* (control) at 24 h and 48 h. (e) The expression levels of *DMR6* on wheat leaves infested with *SmCSP4*‐silenced *S. miscanthi* at 24 h and 48 h. Wheat leaves infested with aphids treated with ds*GFP* were used as control. Three replicates were performed for each treatment. All data shown are mean ± SE. ‘ns’ indicates no significant differences between controls and treatments. Asterisks above bars and lines indicate significant differences between controls and treatments (**P < 0.01; ***P < 0.001; Student's *t* test). (f) Effects of SmCSP4 on binding activity of TaWRKY76 to the promoter of *DMR6* by GUS staining assays. *N. benthamiana* leaves were infiltrated with *Agrobacterium* harbouring *35S*:*GUS*, *proDMR6*:GUS, *35S*:*TaWRKY76‐GFP*, *35S*:*SmCSP4‐GFP* alone, or co‐infiltrated with various *Agrobacterium* mixture. Leaf disk was stained using GUS staining at 2 days post‐infiltration, and then decolorized with 70% ethanol. (1) *35S*:*GUS*; (2) *proDMR6*:*GUS*; (3) *proDMR6*:*GUS*+*35S*:*TaWRKY76‐GFP*; (4) *proDMR6*:*GUS*+*35S*:*TaWRKY76‐GFP*+*35S*:*SmCSP4‐GFP*; (5) *35S*:*SmCSP4‐GFP*; (6) *35S*:*TaWRKY76‐GFP*. The experiments were conducted with six biological replicates. Scale bar = 2 mm. The values are presented as mean ± SE. Different letters above the bars indicate significant difference among treatments (*P* < 0.05, one‐way ANOVA followed by Duncan's multiple range test). (g) The effect of SmCSP4 on TaWRKY76 activated *DMR6* expression in *N. benthamiana* leaves using dual‐luciferase reporter assays. CaMV*35S*‐empty vector was used for normalization between samples. Data are means of three biological replicates (±SE). Different letters above the bars indicate significant difference among treatments (*P* < 0.05, one‐way ANOVA followed by Duncan's multiple range test).

*DMR6* was significantly down‐regulated in *TaWRKY76*‐silenced wheat plants as described above, and two potential WRKY binding regions were also identified in the promoter sequence of *DMR6* gene (Figure S6). To investigate the transcriptional activity of TaWRKY76 in regulating *DMR6* gene expression, we performed Y1H assays. In the transcriptional activity assay, yeast cells harbouring pGBKT7‐*TaWRKY76* could grow on SD/‐Trp/‐Leu/‐His medium, indicating that TaWRKY76 acts as a transcriptional activator (Figure 9a). Yeast cells co‐transformed with AD and pHIS2‐*proDMR6* or with AD‐*TaWRKY76* and pHIS2‐*proDMR6* could grow on SD/‐Trp/‐His/‐Leu medium. However, on SD/‐Trp/‐His/‐Leu medium containing 50‐mm 3‐AT, positive controls and yeast cells containing AD‐*TaWRKY76* and pHIS2‐proDMR6 could grow well, whereas those carrying AD and pHIS2‐*proDMR6* failed to grow (Figure 9b), indicating that *DMR6* is transcriptionally regulated by TaWRKY76. Dual‐luciferase assay was then performed to confirm the binding of TaWRKY76 to the *DMR6* promoter. The promoter sequence of *DMR6* was fused with the *LUC* reporter gene to construct the *proDMR6*‐*LUC* vector, and the *TaWRKY76* CDS was cloned into pGreenII62‐SK to generate the effector plasmid. The relative LUC/REN ratio significantly increased in *N. benthamiana* leaves infiltrated with pGreenII0800‐*proDMR6*‐*LUC* and pGreenII62‐*TaWRKY76*‐*SK* at a rate nearly sevenfold greater than that of control plants, suggesting that TaWRKY76 specifically binds to the *DMR6* promoter *in vivo* (Figure 9c).

As shown in Figure 9d,e, when SmCSP4 was delivered into wheat leaves, the expression levels of *DMR6* were significantly down‐regulated. Moreover, the transcript levels of *DMR6* were significantly higher in wheat leaves infested with *SmCSP4*‐knockdown aphids when compared to controls. These results indicated that SmCSP4 was involved in the regulation of *DMR6* expression in wheat. To further examine whether the transcriptional activation of *DMR6* by TaWRKY76 was affected by SmCSP4, *β*‐Glucuronidase (GUS) staining and dual‐luciferase reporter assays were performed. In the GUS staining assay, no blue colour was observed in *N. benthamiana* leaves infiltrated with *35S*:*TaWRKY76* or *35S*:*SmCSP4*, *N. benthamiana* leaves co‐infiltrated with *proDMR6*:*GUS* and *35S*:*TaWRKY76‐GFP* stained more intensely than those infiltrated with *proDMR6*:GUS, suggesting that TaWRKY76 acts as an activator of *DMR6*. However, GUS staining was significantly lighter in leaves co‐infiltrated with *35S*:*SmCSP4*, *proDMR6*:*GUS*, and *35S*:*TaWRKY76* (Figure 9f). To verify the histochemical staining, the GUS activity was examined in the injection area of each leaf. The results were consistent with the GUS phenotypic observations (Figure 9f).

Furthermore, the promoter region of *DMR6* was fused with the *LUC* reporter gene to construct the *proDMR6*‐*LUC* vector, and the CDS of *SmCSP4* or *TaWRKY76* was cloned into pGreen II 62‐SK to generate effector plasmids (Figure 9g). The interaction of TaWRKY76 with proDMR6‐LUC led to 6.2‐fold increase in the relative LUC/REN ratio in *N. benthamiana* leaves (Figure 9g). However, the LUC/REN ratio was significantly reduced to only 2.3‐fold when SmCSP4 was co‐infiltrated with TaWRKY76 and proDMR6‐LUC. In summary, these results indicate that TaWRKY76 is a transcriptional activator of *DMR6*, but SmCSP4 secreted by aphids reduces the transcriptional activation activity of TaWRKY76 on *DMR6* expression.

## Discussion

The salivary elicitors of aphids, especially those that attack cereal crops, and their mechanism of eliciting plant defence responses have rarely been characterized to date. In this study, SmCSP4, a salivary protein of *S. miscanthi* was found to be secreted into plant cells during aphid feeding, and the expression level of *SmCSP4* was significantly up‐regulated during aphid infestation on wheat plants. Previous studies found that Mp10, the CSP4 homologue of *M. persicae*, was detected in cytoplasm and nucleus of *N. benthamiana* (Rodriguez *et al*., 2014). However, the subcellular localization assay in our study proved that SmCSP4 is translocated to the plant cell nucleus. Furthermore, the subcellular localization of SmCSP4 with mutant NLS changed that were detected in both cytoplasm and nucleus of cells, confirming the nuclear localization of SmCSP4. SmCSP4 with NLS mutations could also be detected in plant nucleus, it is proposed that SmCSP4 is a small protein which could be positively diffused into plant nucleus when NLS was mutated.

*Sitobion miscanthi* is an oligophagous insect and targets wheat as its main host (Blackman and Eastop, 2000). To verify the roles of SmCSP4 in regulating wheat defence, *SmCSP4* was delivered into wheat cells using the EtHAn‐mediated delivery system, which has been successfully applied to study the functions of effectors of the wheat stripe rust fungus *Puccinia striiformis* f. sp. *tritici* (Bai *et al*., 2021; Cheng *et al*., 2017; Upadhyaya *et al*., 2014; Xu *et al*., 2019b). The delivery of *SmCSP4* in wheat leaves induced H_2_O_2_ accumulation and increased the number of callose deposits. Moreover, the expression levels of SA pathway‐associated genes *PAL* and *PR1* were significantly up‐regulated in *SmCSP4*‐infiltrated wheat leaves, whereas the expression of JA‐responsive genes FAD was inhibited. In addition, the SA content of wheat leaves was significantly increased after *SmCSP4* infiltration, whereas the JA content showed no significant change. Additionally, we found that SmCSP4 with NLS mutant activated lower SA levels, indicating that NLS domain is vital for the eliciting activity of SmCSP4.

RNAi has been commonly used to characterize the function of hemipteran genes encoding salivary proteins (Guo *et al*., 2020; Xu *et al*., 2019a). Recently, nanocarrier‐mediated RNAi has become a practical strategy for silencing target genes in insects and holds great potential as a pest control strategy (Niu *et al*., 2021; Yan *et al*., 2020). In this study, to further examine the potential roles of *SmCSP4* in activating SA‐mediated plant defence, the *SmCSP4* gene was silenced after the application of the nanocarrier‐mediated transdermal dsRNA delivery system. Our results indicated that the *SmCSP4* expression significantly decreased after the application of *dsSmCSP4*/nanocarriers/detergent, and its transcript levels reduced by more than 60% compared with the controls, suggesting that nanocarrier‐mediated RNAi is an effective strategy for testing gene function in aphids. After the silencing of *SmCSP4*, lower SA levels were activated in wheat leaves. Therefore, we concluded that SmCSP4 induces SA‐mediated wheat defence responses. However, aphid fecundity was significantly decreased after the silencing of *SmCSP4*, suggesting that *SmCSP4* is essential for aphid fitness on host plants. Previous studies have demonstrated that chemosensory proteins (CSPs) are related to insect xenobiotic detoxification. CSP2 and CSP3 from whitefly *B. tabaci* show a high binding affinity to α‐pentyl‐cinnamaldehyde, which is a common chemical in plant oil with toxic effects upon direct contact (Liu *et al*., 2016). Recently, the high‐affinity binding of pyrethroid insecticides revealed that the leg‐enriched sensory appendage protein, SAP2, exhibited pyrethroid resistance to malaria mosquito *Anopheles gambiae* (Ingham *et al*., 2020). CSP5 protein of *A. gossypii* and the CSP4 and CSP6 proteins of *R. padi* play significant roles in improving aphid resistance to insecticides (Li *et al*., 2021; Peng *et al*., 2021). A previous study showed that the contents of 2,4‐dihydroxy‐7‐methoxy‐(2H)‐1,4‐benzoxazin‐3(4H)‐one (DIMBOA), a toxic plant secondary metabolite in aphid‐resistant wheat variety Zhong4wumang were significantly higher than those in aphid susceptible wheat varieties (Liu *et al*., 2002). In our study, SmCSP4 was expressed higher on Zhong4wumang than on susceptible one Mingxian169. It is speculated that *S. miscanthi* secreted more SmSCP4 to bind specific toxic metabolites in resistant wheat to improve the fitness on hosts. Meanwhile, a coevolutionary arm race between aphid and plant may occur that plant can perceive the secreted SmCSP4 to active stronger SA‐mediated defence response to enhance plant resistance against aphids. Whether SmCSP4 is involved in insecticide resistance or other physiological processes of *S. miscanthi* is worthy of further investigation.

The survival rate and fecundity of *S. miscanthi* were significantly decreased after feeding on *SmCSP4*‐infiltrated wheat leaves, and its feeding behaviour was also negatively affected, with shorter durations of phloem ingestion phases. It has been proposed that the SA signalling defence pathway elicited by SmCSP4 decreases aphid performance. Consistently, we found that the exogenous application of SA to wheat plants induced plant defence and reduced *S. miscanthi* fecundity. Our findings are consistent with those of previous studies showing that SA can contribute to plant resistance against aphids. The accumulation of SA and exogenous application of SA analogues in tomato and *Arabidopsis* are associated with reduced aphid performance and feeding preferences (Avila *et al*., 2012; Boughton *et al*., 2006; Cooper *et al*., 2004). Elimination of SA accumulation increased aphid longevity on tomato plants expressing *NahG* and completely abolished aphid resistance in plants carrying the *Mi‐1* resistance gene, demonstrating that SA is essential for potato aphid resistance (Li *et al*., 2006).

Interestingly, the expression of the *CSP4* homologues of *M. persicae*, *A. pisum* and *A. gosspyii* induced callose deposition and SA‐responsive defence responses in wheat, resulting in a significant reduction in aphid fecundity. These findings demonstrated the conserved role of aphid CSP4 homologues in activating plant immunity. *M. euphorbiae* effector Me10 and its homologue in *A. gossypii*, Ag10k, have been proven to promote aphid virulence in a conserved pathway by interacting with TFT7 *in planta* and interfering with the function of the TFT7 immune complex (Chaudhary *et al*., 2019). Sequence alignment analysis revealed that the CSP4 homologues of four different aphid species exhibit high sequence identity and conserved functional domains. Therefore, whether the regulatory mechanisms involved in activating plant defence are conserved among these homologues should be investigated in the future.

WRKY transcription factors comprise one of the largest families of transcriptional regulators exclusively found in plants and play important roles in plant resistance to various pathogens and insect pests. Several WRKYs have been shown to interact with the salivary effectors of insects to regulate host plant immunity. For example, the effector Bsp9 of whitefly (*Bemisia tabaci*) interacts with Arabidopsis WRKY33 and interferes with the interaction between WRKY33 and MPK6, thus suppressing the plant defence responses (Wang *et al*., 2019). Vitellogenin protein secreted by small brown planthopper (*Laodelphax striatellus*) directly interacts with the rice (*Oryza sativa*) transcription factor OsWRKY71 and weakens H_2_O_2_‐mediated plant defence (Ji *et al*., 2021). In our study, full‐length SmCSP4 (containing the NLS) interacted with TaWRKY76, which has not been functionally characterized in wheat, in the nucleus. A previous study reported that the CathB3 salivary elicitor of *M. persicae* suppresses aphid feeding by interacting with plant EDR1‐like in phloem and triggering the accumulation of reactive oxygen species (Guo *et al*., 2020). To the best of our knowledge, salivary elicitors targeting WRKY transcription factors and eliciting plant immunity have not been reported in aphids to date.

Yeast transcriptional activation verification assays indicated that TaWRKY76 possesses transcriptional activation activity. Furthermore, silencing of *TaWRKY76 via* VIGS up‐regulated the SA content of wheat plants, implying that TaWRKY76 is involved in the inhibition of SA biosynthesis or the promotion of SA degradation as a transcriptional activator. In Arabidopsis, DMR6 encodes a putative 2OG‐Fe (II) oxygenase, which is involved in SA hydroxylation and is required for the susceptibility to downy mildew through the regulation of SA degradation (Van Damme *et al*., 2008; Zhang *et al*., 2017). In apple (*Malus domestica*), WRKY17 is essential for SA degradation and fungal disease susceptibility by directly binding to the promoter of *DMR6* and promoting its expression (Shan *et al*., 2021). But how SA biosynthesis is transcriptionally regulated by TaWRKY76 is hitherto unknown. Herein, the expression of TaWRKY76 increased *LUC* expression when transiently transformed in *N. benthamiana* leaves under the control of the *DMR6* promoter, confirming that TaWRKY76 directly activates *DMR6* expression *in vivo*. The *DMR6* orthologs were highly up‐regulated in the aphid‐susceptible wheat variety after infestation by *Puccinia graminis* f. sp. *tritici* (Henningsen *et al*., 2021). Given its essential role in plant disease susceptibility, the loss of function of *DMR6* by RNAi and genome editing in crops are promising strategies for controlling plant pathogens. For instance, mutations in the Arabidopsis *DMR6* gene confer resistance to hemibiotrophic pathogens *Pseudomonas syringae* and *Phytophthora capsici* as well as *sldmr6‐1* tomato mutants display enhanced broad‐spectrum resistance against different classes of pathogens, such as bacteria, oomycetes, and fungi (Thomazella *et al*., 2021; Zeilmaker *et al*., 2015). We also found that silencing of *TaWRKY76* induced SA accumulation and caused detrimental effects on aphid performance and feeding behaviour, suggesting that *TaWRKY76* is a susceptible gene in wheat. Previously, the CRISPR‐Cas9‐mediated inactivation of the susceptible gene *TaPsIPK1* in wheat targeted by *P. striiformis* f. sp. *tritici* (Pst) effector PsSpg1 conferred broad‐spectrum resistance against Pst without affecting the important agronomic traits of wheat plants (Wang *et al*., 2022). Therefore, susceptible genes *TaWRKY76* and *DMR6* could be promising targets for engineering wheat plants with broad‐spectrum resistance against aphids and pathogens.

In GUS staining and dual‐luciferase assays, SmCSP4 secreted by aphids could suppress the binding of TaWRKY76 to the *DMR6* promoter, which decreased gene expression. The SmCSP4‐induced reduction in *DMR6* expression level may subsequently cause an increase in the SA content and greater plant resistance against aphids. Similarly, a previous study reported that the salivary effector vitellogenin of *L. striatellus* negatively affects the transcriptional activity of the interacting protein OsWRKY71 and influences the expression of *OsGF14b*, suppressing H_2_O_2_‐mediated plant defence (Ji *et al*., 2021). However, further research is warranted to elucidate the molecular mechanism of SmCSP4‐mediated inhibition of the transcription activation activity of TaWRKY76.

**Figure 10:**
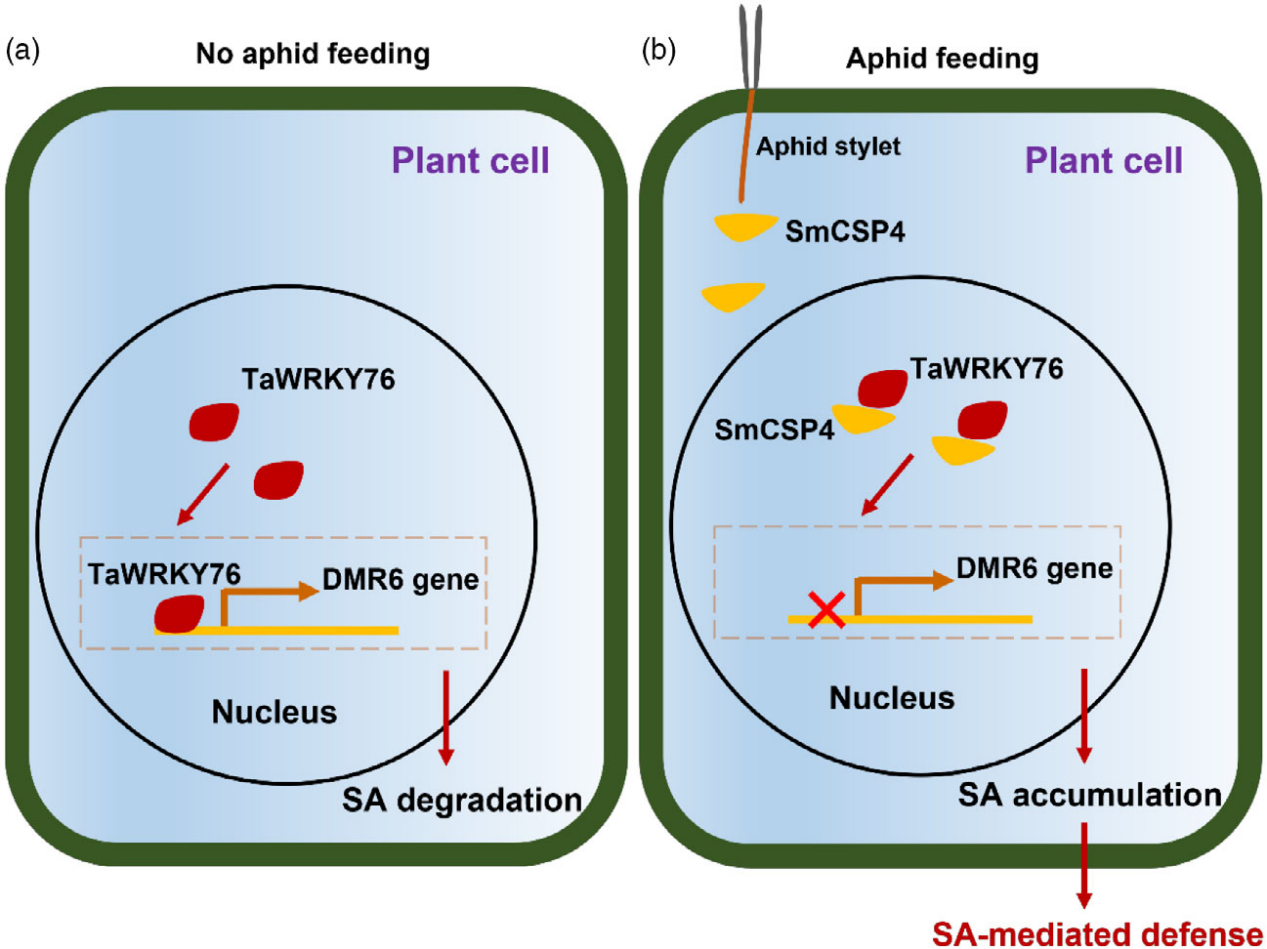
A schematic summary of the roles of SmCSP4 secreted by *Sitobion miscanthi* during aphid–wheat interactions. (a) In wheat plants, TaWRKY76 can bind to the promoter of DMR6 and induce its gene expression as transcriptional activator, causing SA degradation. (b) During the probing and feeding of *Sitobion miscanthi*, salivary protein SmCSP4 is secreted into plant cells which can be interacted with TaWRKY76 in nucleus. Meanwhile, the regulation process of TaWRKY76 on DMR6 gene expression is inhibited by interacting with SmCSP4, which resulted in increases of SA accumulation and enhanced SA‐mediated plant resistance against *S. miscanthi*.

In summary, our study demonstrates that the salivary elicitor SmCSP4 of *S. miscanthi* activates SA‐mediated wheat defence responses by interacting with TaWRKY76. SmCSP4 reduced the transcriptional activity of TaWRKY76 to regulate the expression of the SA degradation gene *DMR6*, resulting in enhanced SA levels and greater plant immunity against aphids (Figure 10).

## Experimental procedures

### Aphids and plants

Winter wheat (*Triticum aestivum*) cultivars Mingxian169 (aphid‐susceptible) and Zhong4wumang (aphid‐resistant) were used in this study (Xu *et al*., 2011). Seeds were incubated in sterilized Petri dishes containing distilled water for 3–4 days at 25 ± 1 °C to induce germination. Healthy seedlings were carefully transferred into plastic pots containing organic soil (peat: vermiculite = 3 : 1) and grown in a climate chamber at 20 ± 1 °C under 16 h light/8 h dark photoperiod for 12 days (i.e., until reaching the two‐leaf stage). Tobacco plants *Nicotiana tabacum* and *N. benthamiana* were grown in an environmentally controlled greenhouse at 23 ± 1 °C under 16 h light/8 h dark photoperiod. After 4–5 weeks, the *N. benthamiana* plants were used in transient transformation experiments using *Agrobacterium tumefaciens*.

Clones of *S. miscanthi*, *A. pisum* and *A. gossypii* were initially established from a single aphid collected from a field in Langfang City, Hebei Province, Northern China, and clones of *M. persicae* were established from a single aphid collected from a tobacco field in Kunming City, Yunnan Province, Southwestern China. No pesticides were applied on the collection sites. All aphid clones were maintained under laboratory conditions (16 h light/8 h dark photoperiod and 20 ± 1 °C) on the corresponding host plants, including wheat, broad bean *Vicia faba*, cotton *Gossypium hirsutum*, and *N. tabacum*.

### Sequence analysis

The signal peptide of SmCSP4 was identified by using SignaIP 5.0 (https://services.healthtech.dtu.dk/service.php?SignalP‐5.0). The localization of the SmCSP4 in cells was predicted using LOCALIZER (http://localizer.csiro.au/index.html) and WoLF PSORT II (https://www.genscript.com/wolf‐psort.html). Multiple sequence alignments of CSP4 were performed using Clustal Omega (https://www.ebi.ac.uk/Tools/msa/clustalo/). Phylogenetic analysis of amino acid sequences of CSPs was performed with the maximum likelihood method using MEGA7 software (https://www.megasoftware.net/). The amino acid sequences of CSPs were listed in Table S1. The binding sites of transcriptional factor TaWRKY76 on the promoter sequence of *DMR6* were predicted using JASPAR (https://jaspar.genereg.net/analysis).

### Subcellular localization of *SmCSP4* in *N. benthamiana*

To perform subcellular localization assays, the *SmCSP4* CDS excluding the signal peptide (*SmCSP4*) and *SmCSP4* with mutant nuclear localization signal (*SmCSP4* ^*NLSm*^) were cloned into the pCAMBIA1300 vector using the In‐Fusion Cloning Kit (Takara, Japan) according to the manufacturer's protocols. An A to C single base mutation was introduced in base 422 of the coding sequence of *SmCSP4* ^*NLSm*^. *A. tumefaciens* GV3101 cells carrying the pCAMBIA1300:*SmCSP4‐GFP*, pCAMBIA1300:*SmCSP4* ^*NLSm*^ *‐GFP* or pCAMBIA1300:*GFP* (control) construct were resuspended in infiltration buffer (10 mm MgCl_2_, 10 μm acetosyringone, 10 mm 2‐(N‐morpholino) ethanesulfonic acid [pH 5.6]) at a final optical density OD_600_ of 0.5 and then infiltrated into 4‐week‐old *N. benthamiana* leaves as described previously (Bos *et al*., 2010). Then, GFP signal was detected *via* Confocal Laser Scanning Microscope (LSM700, Zeiss) at an excitation wavelength of 488 nm at 24 h post‐infiltration (hpi). DAPI was used as a counterstain to observe the nuclei.

### Delivery of SmCSP4 into wheat via the type III secretion system (T3SS)

The *SmCSP4* or *SmCSP4* ^*NLSm*^ sequence was cloned into the pEDV6 vector *via* Gateway recombination (Thermo Fisher, USA) according to the manufacturer's protocols, and the resultant construct (pEDV6:*SmCSP4* or pEDV6:*SmCSP4* ^*NLSm*^) was transformed into *Pseudomonas fluorescens* strain EtHAn *via* electroporation. The recombinant EtHAn strain carrying pEDV6:*SmCSP4* or pEDV6:*SmCSP4* ^*NLSm*^ was grown in KB liquid medium (25 μg/mL rifampicin, 30 μg/mL gentamicin) for 24 h, and then cells were washed with 10 mm MgCl_2_ solution for three times and resuspended in 10 mm MgCl_2_ solution. EtHAn cell suspension (OD_600_ = 1.0) was infiltrated into the second leaf of 12‐day‐old wheat seedlings (*var*. Zhong4wumang) at the two‐leaf stage using a needleless syringe. Wheat leaves infiltrated with 10 mm MgCl_2_ solution or EtHAn carrying pEDV6:*DsRed* or pEDV6:*AvrRpt2* were used as blank, negative, and positive controls respectively (Xu *et al*., 2019b). The infiltrated plants were grown and maintained in a climate chamber at 20 ± 1 °C for 2 days.

### Western blot

Wheat leaves (12‐day‐old, var. Mingxian 169) were infested with 600 *S. miscanthi* for 2 days. Aphids were then removed carefully from each leaf using soft brush and the leaves were immediately washed with distilled water for 3 times. Western blot was performed to detect SmCSP4 protein in *S. miscanthi* whole body, aphid‐infested wheat leaves and aphid‐uninfested leaves using purified SmCSP4 rabbit polyclonal antibody (ABclonal, China). Wheat leaves without aphid infestation were used as control.

*SmCSP4*‐or SmCSP4^NLSm^‐expressed wheat leaves were collected at 2hpi, and the SmCSP4 or SmCSP4^NLSm^ protein was also detected by Western blot using anti‐SmCSP4 (ABclonal, China) or anti‐GFP polyclonal antibody (Cat. No. D110008, Sango Biotech, China). Protein was extracted from aphid whole body and plant leaves using RIPA Lysis Buffer for Western and IP (Beyotime, China) and then quantified using BCA protein assay kit (Bio‐Rad, USA). Equal amount of protein of each sample was separated by 10% SDS‐PAGE and transferred onto a Polyvinylidene Fluoride (PVDF) membrane. PVDF membrane was blocked with 5% BSA for 2 h at room temperature and then incubated with purified polyclonal antibody (1 : 1000 dilution) overnight. Anti‐actin polyclonal antibody for plant (Cat. No. D110007, Sango Biotech, China) and insect (Cat. No. bs‐8778R, Beijing Biosynthesis Biotechnology Co., Ltd. China) were used as loading controls for Western blot analysis. The antigen–antibody complexes were visualized using a goat anti‐rabbit IgG‐conjugated horseradish peroxidase (HRP) antibody (Cat. No. sc‐2054, Santa Cruz Biotechnology, Inc., USA) at a 1 : 10 000 dilutions. Clarity ECL solution (Bio‐Rad, USA) was added for detection, and the chemiluminescence signal was then captured with a ChemiDoc XRS^+^ imaging system.

### RNAi using the nanocarrier‐mediated delivery system

Double‐stranded RNA (dsRNA) of *SmCSP4* was prepared using TranscriptAid T7 High‐Yield Transcription Kit (Thermo Fisher Scientific, CA) according to the manufacturer's protocols, with the primers listed in Table S2. The dsRNA of *SmCSP4* or *GFP* was mixed with the SPc nanocarrier at a 1 : 1 ratio (w/w). Then, 0.05% detergent (surfactant and softened water) was added to the solution to reduce the surface tension of the hydrophilic nanocomplexes (Li *et al*., 2019; Yan *et al*., 2020). Subsequently, 0.1 μL of the *SmCSP4* dsRNA/nanocarrier/detergent formulation or *GFP* dsRNA/nanocarrier/detergent formulation (control) with a dose of 500 ng/μL dsRNA was dispensed on the notum of apteral adult aphids. After the solution was completely dried, all aphids were carefully transferred onto wheat plants using a soft brush. The dsRNA‐treated aphid samples were collected at 24 or 48 h post‐infestation on wheat and washed three times with distilled water to remove any residues of nanocarrier/detergent. Real‐Time Quantitative PCR (RT‐qPCR) was performed to detect the transcript levels of *SmCSP4*. Each treatment was performed in triplicate, and each replicate contained five dsRNA‐treated aphids.

### Y‐tube olfactometer assay

Behavioural assays were performed using a glass Y‐tube olfactometer (2.7 cm diameter, 10 cm trunk length, and 16.5 cm branch length) as described previously (Zhang *et al*., 2015). The air was purified by passing through granular activated carbon at a flow rate of 0.1 L/min. One arm of the Y‐tube was introduced with EBF (dissolved in hexane at a concentration of 10 ng/μL) or volatiles of whole wheat plants, whereas the other arm (control) was introduced with hexane or clean air, respectively. A Y‐shaped copper wire was placed at the centre of the Y‐tube to facilitate aphid mobility. Aphids treated with ds*SmCSP4*/nanocarrier/detergent, ds*GFP*/nanocarrier/detergent or nanocarrier/detergent (negative control) formulations at 48 h as mentioned above were then placed at the end of the copper wire in the trunk. The number of aphids in each arm exceeded 5 cm was recorded after 10 min. A total of 15 replicates (with 30 apterous aphids per replicate) were conducted for each treatment.

### Detection of callose deposition and H_2_O_2_ accumulation in leaves

Aniline blue staining was performed to visualize callose deposition in wheat leaves at 2 days post‐infiltration (dpi), as described previously (Hood and Shew, 1996). Callose deposition was observed and photographed under an Echo Revolve Hybrid Microscope (Echo Laboratories, USA) using a DAPI (4, 6‐diamidino‐2‐phenylindole) filter. The number of callose deposits was counted at 15 different sites (1 mm^2^ area per site) on each infiltrated leaf. Five biological replicates were performed for each treatment.

The accumulation of H_2_O_2_ in wheat leaves was detected *via* DAB staining according to the protocol described previously (Zhao *et al*., 2018). The stained leaves were imaged using a Canon digital camera or Olympus SZX‐16 (Olympus Corporation, Japan). The endogenous H_2_O_2_ levels in wheat leaves were determined using hydrogen peroxide (H_2_O_2_) Content Assay Kit (Sangon Biotech, China) according to the manufacturer's protocols. Six biological replicates for each treatment were conducted, and five leaf samples with the same treatment were pooled as one replicate.

### Analyses of SA and JA contents

Phytohormone contents were examined as described previously (Xiang *et al*., 2011), with minor modifications. Briefly, 0.5 g of wheat leaves infested with twenty apterous *S. miscanthi* adults or infiltrated with MgCl_2_, *P. fluorescens* EtHAn carrying pEDV6:*DsRed*, pEDV6:*SmCSP4*, pEDV6:*SmCSP4* ^*NLSm*^, pEDV6:*ApCSP4*, pEDV6:*MpCSP4* or pEDV6:*AgCSP4* was harvested at 2 dpi and homogenized in liquid nitrogen mixed with labelled internal standards (^2^D_4_‐SA, ^2^D_6_‐JA). Then, 10 mL of ethyl acetate was added to the sample. This was followed by ultrasound extraction for 20 min and centrifugation at 14 000 *g* for 20 min at 4 °C. After vacuum‐drying, the final extracts were re‐dissolved in methanol and filtered through a 0.2 μm nylon filter. The SA and JA contents in wheat leaves were measured *via* liquid chromatography–tandem mass spectrometry (LC–MS/MS). At least five biological replicates were performed.

### RT‐qPCR

Total RNA isolation and RT‐qPCR assays were performed as described previously (Zhang *et al*., 2022). Briefly, total RNA was extracted from aphid tissues, tobacco or wheat leaves using TRIzol Reagent (Thermo Fisher, USA) according to the manufacturer's protocols. First‐strand cDNA synthesis was conducted using the HiScript III First‐Strand cDNA Synthesis Kit (+gDNA wiper) (Vazyme, China), and RT‐qPCR was performed on the ABI Prism 7500 Real‐Time PCR System using SYBR Premix Ex Taq Kit (TaKaRa, Japan) and sequence‐specific primers (Table S2). Three replicates were conducted for each treatment, and each replicate contains three technical replicates.

### Aphid bioassays

To investigate the effects of gene silencing on *S. miscanthi* performance, 10 dsRNA‐treated apterous adult aphids were transferred onto wheat leaves (*var*. Zhong4wumang) and confined using a clip cage (2.5 cm × 2.5 cm × 2.5 cm). The number of surviving aphids was counted every other day for 8 days, and a total of 15 replicates were performed for each treatment. Five apterous adult aphids were also treated with dsRNA as mentioned above, and the number of nymphs produced by aphids was recorded daily for 5 days. Aphids treated with ds*GFP* were included as a control. A total of 15 replicates were performed for each treatment.

After *P. fluorescens* EtHAn infiltration, exogenous SA application at 2 days, or *TaWRKY76* silencing, 10 apterous adult aphids were transferred onto the treated wheat leaves (*var*. Zhong4wumang) and confined using clip cages as described above. The number of surviving aphids was counted every other day for 8 days, and a total of 15 replicates were performed for each treatment. Five apterous adult aphids were transferred onto the treated wheat leaves as mentioned above, and the number of nymphs produced by aphids was recorded daily for 5 days. Ten newborn nymphs were reared on wheat leaves infiltrated with the *SmCSP4* delivering or *TaWRKY76* silencing construct, and each aphid was weighed on a microbalance (Sartorius, GPC, Germany) after 7 days of feeding. Fifteen replicates were performed for each treatment. Wheat plants treated with SA solvent, *P. fluorescens* EtHAn carrying pEDV6:*DsRed*, or BSMV::00 were used as control, respectively.

### Assessment of aphid feeding behaviour using electronic penetration graph (EPG)

The feeding behaviour of adult apterous aphids on wheat leaves (*var*. Zhong4wumang) expressed with SmCSP4 or DsRed (control) was recorded continuously for 6 h in a Faraday cage using EPG (DC‐EPG Giga‐8) in an environmentally controlled room (20 °C ± 1 °C, 16 h light/8 h dark photoperiod, 70% relative humidity). EPG waveforms were recorded and analysed using the Stylet+ software (EPG system; www.epgsystems.eu). The characteristics of aphid feeding waveform patterns were identified as previously reported (Prado and Tjallingii, 1994). Ten replicates were conducted for each treatment.

### Y2H assay

*Sitobion miscanthi*‐infested wheat leaves were harvested at 6, 12, 24, 48 and 96 hpi, and cDNA libraries were constructed by OE Biotech. Co., Ltd. (Shanghai, China) using the CloneMiner II cDNA Library Construction Kit (Thermo Fisher, USA) according to the manufacturer's protocols. The CDS of *SmCSP4* corresponding to the mature protein was cloned into the pGBKT7 vector to generate *SmCSP4*‐BD (bait vector), and putative SmCSP4‐interacting proteins were screened using the Matchmaker Gold Yeast Two‐Hybrid System (Clontech, USA) according to the manufacturer's protocols. To further confirm the interaction between SmCSP4 and TaWRKY76, the yeast two‐hybrid (Y2H) gold yeast strain was co‐transformed with *SmCSP4*‐BD and *TaWRKY76*‐AD constructs, and serial dilutions of the transformed cells were plated onto appropriate selective dropout media. Yeast strains co‐transformed with pGBKT7‐53 and pGADT7‐T, pGBKT7‐Lam and pGADT7‐T were set as positive and negative control mating, respectively.

### BiFC assay

The *SmCSP4* sequence and *TaWRKY76* gene were cloned into pSPYNE‐*35S* and pSPYCE‐*35S*, respectively. Protoplasts were isolated from two young leaves of 4‐week‐old *N. benthamiana* plants that were cut in small pieces and vacuum infiltrated with 5% (w/v) cellulase Onozuka R‐10 and 0.4% (w/v) macerozyme R.10 (Yakult, Japan) in 0.4 M mannitol, and incubated for 3 h followed by shaking at 40 rpm in dark. The homogenates were filtered through a 40 μm nylon mesh and resuspended in W5 solution after centrifuge. The cells were finally resuspended in mannitol‐magnesium buffer to get a density of 2 × 10^5^ protoplasts per mL. For transformation, 10 μg of *SmCSP4*‐*nYFP* and *TaWRKY76*‐*cYFP* constructs were co‐transformed into 200 μL of protoplast suspension by using 40% (v/v) *PEG*. The cells were resuspended in 200 μL W1 buffer and incubated at room temperature for 20 h after centrifuging at 200 g for 1 min. Images were captured by a Confocal Laser Scanning Microscope (LSM700, Zeiss). Upon excitation of 488 nm, yellow fluorescent protein fluorescence and chlorophyll autofluorescence were recorded at 493–531 nm and 644–800 nm, respectively. Plant cell nucleus were stained with DAPI for 30 min prior to detection.

### Co‐IP assay

*SmCSP4*‐*HA* and *TaWRKY76*‐*MYC* were co‐expressed in *N. benthamiana* leaves, and total protein was extracted from these leaves using the RIPA Lysis Buffer (Beyotime, China). Protein extracts were centrifuged at 15000 ***g*** at 4 °C for 30 min and the supernatants were incubated with anti‐HA magnetic beads or anti‐MYC magnetic beads (Thermo Fisher, USA) for 3 h at 4 °C. The Co‐IP assay was performed using the Pierce Magnetic HA‐Tag IP/Co‐IP Kit and Pierce Magnetic MYC‐Tag IP/Co‐IP Kit (Thermo Fisher, USA). The immunoprecipitated proteins were detected *via* Western blotting using anti‐MYC (1 : 1000) antibody or anti‐HA antibody (1 : 1000) (antibodies‐online Inc., Germany). The luminescent signal was visualized using BIO‐RAD ChemiDoc™XRS^+^. *N. benthamiana* leaves co‐expressed with *TaWRKY76*‐*MYC* and *HA* were set as controls, and IP with IgG antibody served as negative control.

### Barley stripe mosaic virus (BSMV)‐mediated gene silencing

A *TaWRKY76* fragment was cloned into the BSMV‐*γ* vector. The recombinant vector and the vector containing the tripartite viral genome were linearized and transcribed using the mMESSAGE mMACHINE® T7 Transcription Kit (Thermo Fisher, USA) according to the manufacturer's protocols. Transcripts of BSMV‐α, BSMV‐*β*, and BSMV‐*γ*/BSMV‐*γ*: *phytoene desaturase* (*TaPDS*)/BSMV‐*γ*:*TaWRKY76* were mixed in 1 : 1 : 1 ratio and then added to FES buffer for inoculation. The BSMV:00 and BSMV:*TaPDS* constructs were used as negative and positive controls for BSMV infection, respectively. The treated leaves were collected at 10 dpi for RNA extraction, and silencing efficiency was detected *via* RT‐qPCR. Three replicates were conducted.

### Transcriptional activation assays in yeast

To perform the transcriptional activation assay in yeast, the *TaWRKY76* CDS was introduced into the pGBKT7 vector, as described previously (Shan *et al*., 2021). The primers used in this experiment are listed in Table S2.

### Yeast One‐Hybrid (Y1H) assay

Y1H assay was carried out using the MATCHMAKER One‐Hybrid System (Clontech, CA, USA), according to the manufacturer's protocols. The *TaWRKY76* CDS was cloned into pGADT7‐*Rec2* to generate the prey vector, and the regulatory elements of the *DMR6* promoter (*proDMR6*) were cloned into pHIS2 to construct the reporter (bait) vector. Then, the Y187 yeast strain was co‐transformed with AD‐*TaWRKY76* and pHIS2‐*proDMR6* and cultured on SD/‐Trp/‐Leu/‐His medium supplemented with 50‐mm 3‐amino‐1,2,4‐triazole (3‐AT) to verify the interaction. Yeast cells co‐transformed with pHIS2‐*p53* and pGAD53m served as a positive control.

### Dual‐luciferase reporter assay

The *TaWRKY76* CDS or *proDMR6* sequence were cloned into the pGreenII‐*062SK* and pGreenII0800‐*LUC* vectors, respectively. To assess the effects of SmCSP4 on the regulatory activity of TaWRKY76, the *SmCSP4* sequence was cloned into pGreenII‐062SK to generate the effector plasmid. Then, the *35S*:*TaWRKY76* or *35S*:*SmCSP4* effector plasmid and the *proDMR6* reporter plasmid were co‐transformed into *A. tumefaciens* strain GV3101, which was then infiltrated into *N. benthamiana* leaves (as described above). Firefly luciferase (LUC) and *Renilla* luciferase (REN) activities were measured using the Dual‐Luciferase Assay System (Promega, USA). Six replicates were performed for each treatment.

### β‐Glucuronidase (GUS) staining assay

The *proDMR6* sequence was cloned into pBI121‐*GUS* (which lacks the *35S* promoter) to generate the *proDMR6*:*GUS* construct. The primer sequences used are listed in Table S2. The recombinant vectors were individually transformed into *A. tumefaciens* strain GV3101. Equal volumes of *Agrobacterium* cultures harbouring *proDMR6*:*GUS*+*35S*:*TaWRKY76‐GFP*, *proDMR6*:*GUS*+*35S*:*SmCSP4‐GFP* and *proDMR6*:*GUS*+*35S*:*TaWRKY76‐GFP*+*35S*:*SmCSP4‐GFP* were mixed and infiltrated into *N. benthamiana* leaves. *Agrobacterium* cells harbouring *35S*:*GUS* served as a positive control, whereas those harbouring *proDMR6*:*GUS*, *35S*:*TaWRKY76‐GFP* or *35S*:*SmCSP4‐GFP* alone served as negative controls. The infiltrated sections of leaves were collected at 2 dpi, immersed in the GUS staining solution overnight at 37 °C and destained with 70% ethanol. The treated *N. benthamiana* leaves were also collected to detect the GUS activity 2 days after the infiltration as described previously (Jefferson *et al*., 1987). Each treatment was conducted with six replicates.

### Statistical analysis

All statistical tests were conducted using the SPSS 20.0 software. To compare two independent groups, statistical significance was determined using Student's *t*‐test. When comparing multiple groups, statistical significance was determined *via* one‐way analysis of variance and Duncan's multiple range test. EPG data were analysed by a Mann–Whitney *U* test. *P* values less than 0.05 were considered to be statistically significant.

## Author contributions

JLC and YZ conceived and designed the project; YZ, YF, XBL, JF, HL, QW and YMZ conducted the experiments; all authors analysed the data; FF and JLC revised the manuscript. YZ and JLC wrote the article with contributions from all other authors.

## Conflict of interest

The authors declare no conflict of interest.

## Supporting information


**Figure S1** Sequence analysis of SmCSP4 from *S. miscanthi*.
**Figure S2** Western blot analysis of SmCSP4 or SmCSP4^NLSm^ protein in wheat leaves post‐2 days infiltration.
**Figure S3** Salicylic acid improved wheat resistance against aphids.
**Figure S4** Olfactory responses of *SmCSP4*‐silenced aphids to aphid alarm pheromone (*E*)‐β‐farnesene and wheat plant volatiles.
**Figure S5** Phylogenetic tree constructed by comparing the amino acid sequences of TaWRKY76 with WRKYs identified from *Arabidopsis thaliana*.
**Figure S6** The potential binding sites of transcriptional factor TaWRKY76 on the promoter of *DMR6* gene.
**Table S1** All CSP amino acid sequences used for phylogenetic tree construction.
**Table S2** All primers used in this study.
