# Supplementary material for: SmCSP4 from aphid saliva stimulates salicylic acid‐mediated defence responses in wheat by interacting with transcription factor TaWKRY76
Source: Plant Biotechnol J. 2023 Aug 4;21(11):2389–407. doi: 10.1111/pbi.14139 (PMC10579719; doi:10.1111/pbi.14139)
Supplement: Supplementary file 1 — Figure S1 Sequence analysis of SmCSP4 from S. miscanthi. Figure S2 Western blot analysis of SmCSP4 or SmCSP4NLSm protein in wheat leaves post‐2 days infiltration. Figure S3 Salicylic acid improved wheat resistance against aphids. Figure S4 Olfactory responses of SmCSP4‐silenced aphids to aphid alarm pheromone (E)‐β‐farnesene and wheat plant volatiles. Figure S5 Phylogenetic tree constructed by comparing the amino acid sequences of TaWRKY76 with WRKYs identified from Arabidopsis thaliana. Figure S6 The potential binding sites of transcriptional factor TaWRKY76 on the promoter of DMR6 gene. Table S1 All CSP amino acid sequences used for phylogenetic tree construction. Table S2 All primers used in this study. [file PBI-21-2389-s001.docx]

**SmCSP4 from aphid saliva stimulates wheat defense responses by interacting with transcription factor TaWKRY76**

Yong Zhang^1^, Yu Fu^2^, Xiaobei Liu^1^, Frédéric Francis^3^, Jia Fan^1^, Huan Liu^1^, Qian Wang^1,4^, Yu Sun^1^, Yumeng Zhang^1,5^, Julian Chen^1*^

^1^ State Key Laboratory for Biology of Plant Diseases and Insect Pests, Institute of Plant Protection, Chinese Academy of Agricultural Sciences, Beijing, 100193, P.R. China

^2^ PHIM Plant Health Institute, Univ Montpellier, INRAE, CIRAD, Institut Agro, IRD, 34000 Montpellier, France

^3^ Functional and Evolutionary Entomology, Gembloux Agro-Bio Tech, University of Liège, Gembloux, B-5030, Belgium.

^4^ Department of Entomology, College of Plant Protection, China Agricultural University, Beijing 100193, P.R. China

^5^ College of Plant Health and Medicine, Qingdao Agricultural University, Qingdao, 266109, P.R. China

Correspondence: [chenjulian@caas.cn](mailto:chenjulian@caas.cn)

ORCIDs:

Yong Zhang: 0000-0003-4134-2405

Julian Chen: 0000-0001-9192-955X

**Supplementary Information**


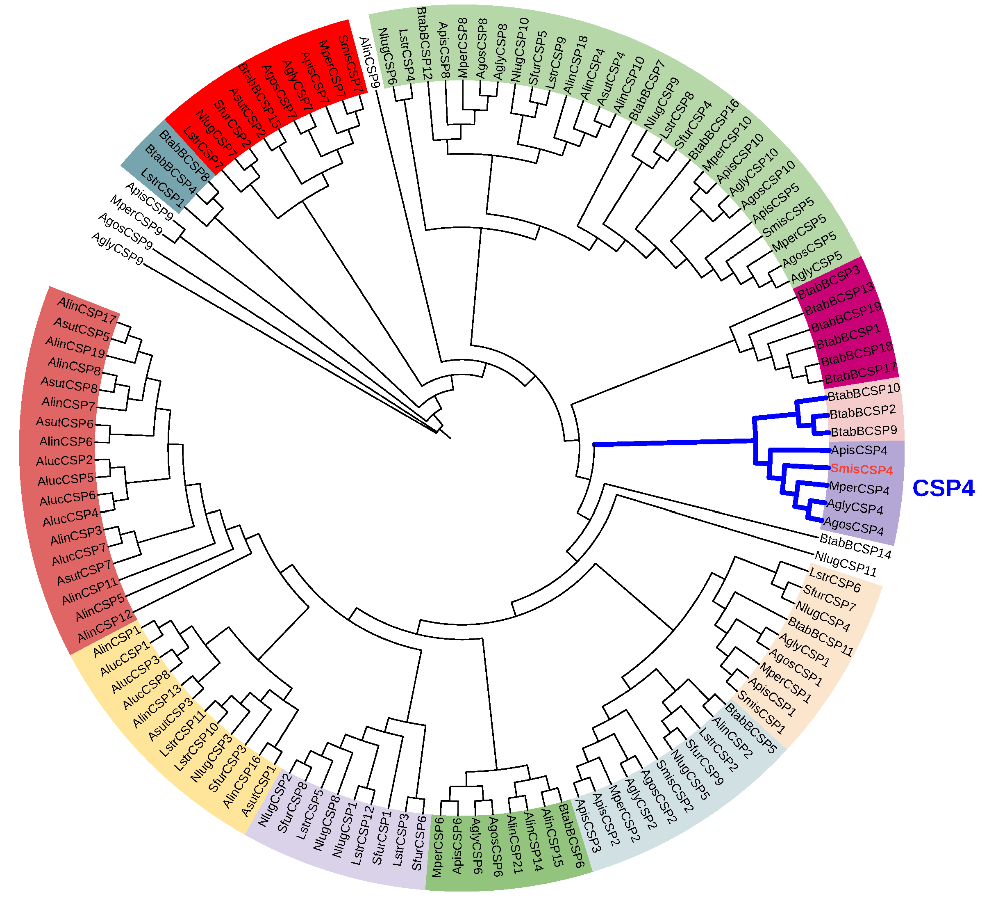


**A**

**B**


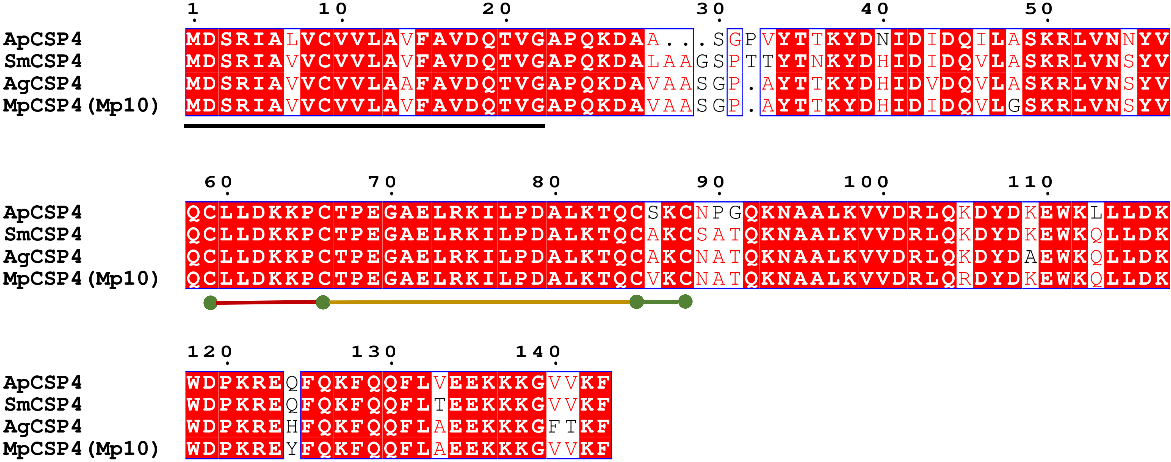


**Figure S1.** Sequence analysis of SmCSP4 from *S. miscanthi*. (A) Phylogenetic tree constructed by comparing the amino-acid sequences of CSPs from other hemipterans species. Abbreviation for various species: *Adelphocoris lineolatus* (Alin), *Apolygus lucorum* (Alu), *Adelphocoris suturalis* (Asut), *Nilaparvata lugens* (Nlug), *Sogatella furcifera* (Sfur), *Laodelphax striatellus* (Lstr), *Stiobion miscanthi* (Smis), *Aphis gossypii* (Agos), *Myzus persicae* (Mper), *Acyrthosiphon pisum* (Apis), *Aphis glycines* (Agly), and *Bemisia tabaci* B biotype (BtabB). Bootstrap values = 1000. (B) Multiple sequence alignment of SmCSP4 protein and orthologs from other three aphid species including *M.persicae* (MpCSP4), *A. pisum* (ApCSP4) and *A. gossypii* (AgCSP4). Red shades indicate identical amino acids. Black underline indicates the signal peptide sequence, and color underlines indicate conserved CX_6_CX_18_CX_2_C domains for CSPs. Expression levels of *SmCSP4* in different tissues of *Sitobion miscanthi*.


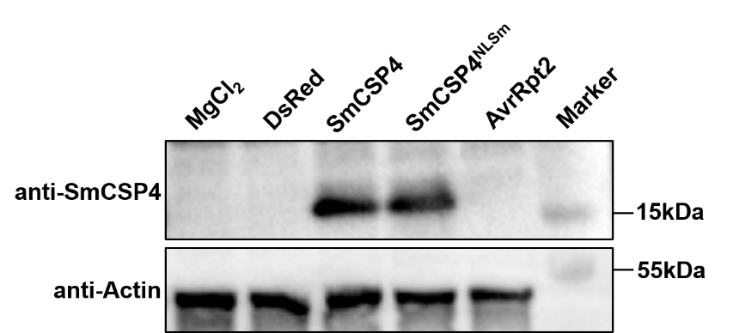


**Figure S2.** Western blot analysis of SmCSP4 or SmCSP4^NLSm^ protein in wheat leaves post 2 days infiltration. Anti-SmCSP4 polyclonal antibody was used to detect SmCSP4 protein in levaes. Plant β-Actin was used as a loading control and detected with anti-actin polyclonal antibody.

**A**


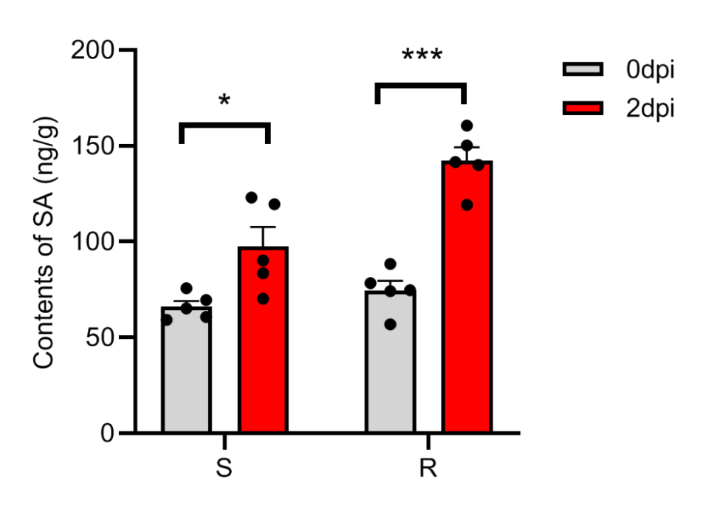


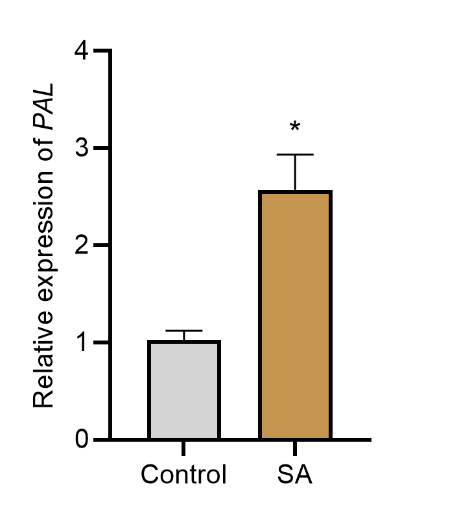

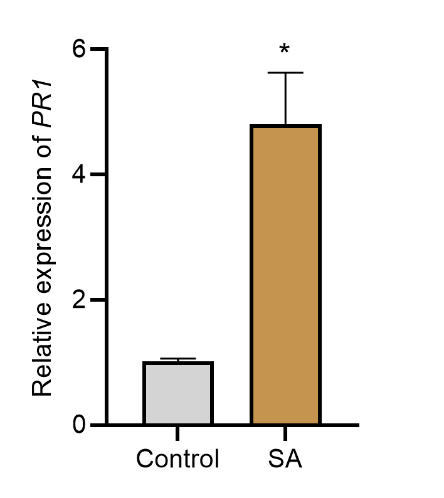


**B**


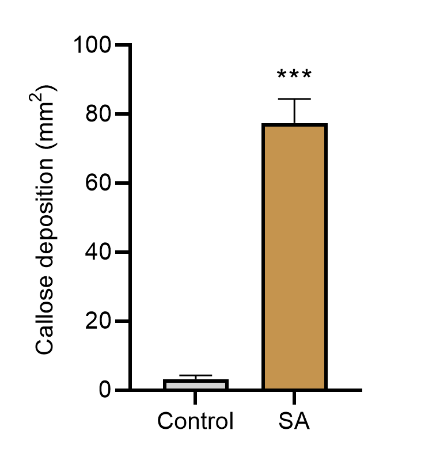


**C**


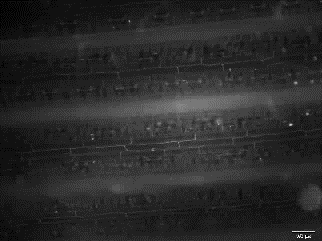

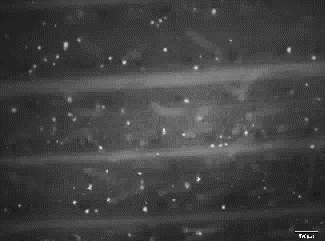


**Control**

**SA**

**D**


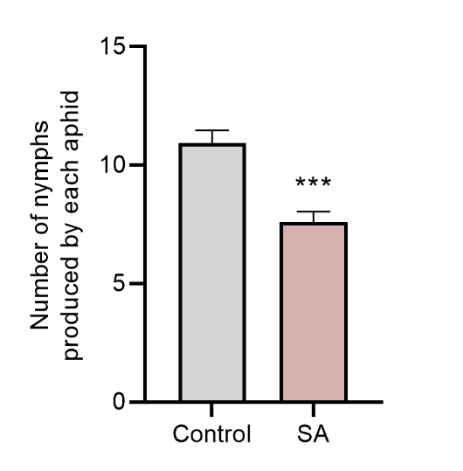

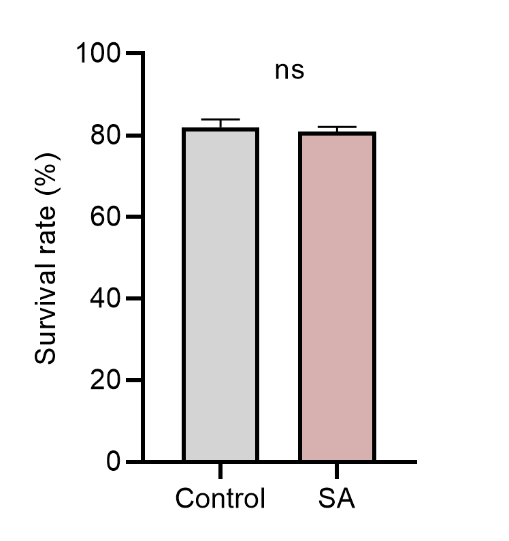


**Figure S3.** Salicylic acid improved wheat resistance against aphids. (A) Changes in endogenous SA contents in leaves of aphid-susceptible (S) and aphid-resistant (R) wheat varieties at 2 days of *S. miscanthi* infestation. The experiment was performed with five replicates. Data are shown as mean ± SE. Different letters above the bars indicate significant differences among treatments (*P* < 0.05, One-way ANOVA followed by Duncan’s multiple range test). (B) Relative expression levels of SA-associated defense genes *PAL* and *PR1* in wheat leaves at 2days post application of SA solution by RT-qPCR. *β-Actin* was used as internal reference gene. Three replicates were used for analysis. (C) Examination of callose deposition in the wheat leaves spayed with SA solution at 2 days using aniline blue staining. Pictures were captured using epifluorescence microscopy. Scale bar=100 μm. (D) Survival rate of *S. miscanthi* and number of nymphs produced by each aphid after feeding on SA treated wheat leaves. Fifteen replicates were conducted for each treatment. All data are presented as the mean ± SE in the boxplot. Asterisks above the bars indicate significant differences between controls and treatments (**P* < 0.05; ****P* < 0.001; Student’s t test).

**
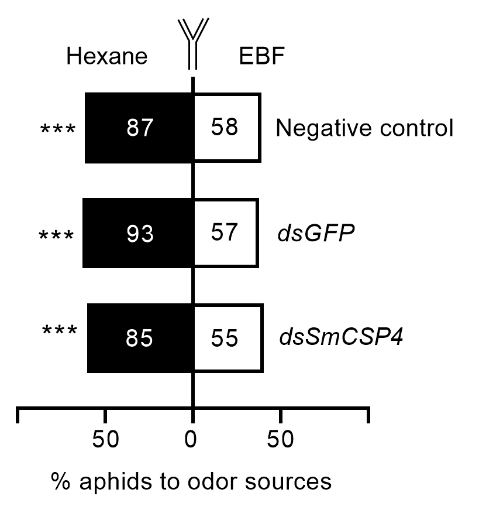

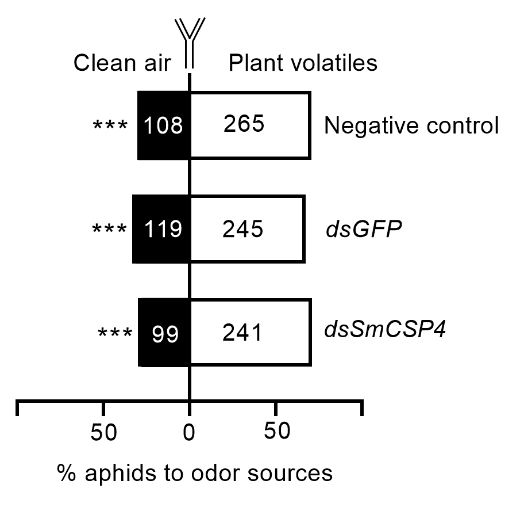
**

**Figure S4.** Olfactory responses of *SmCSP4*-silenced aphids to aphid alarm phenomenon (*E*)-β-farnesene and wheat plant volatiles. White column represents hexane or clean air (control). Aphids treated with ds*GFP* nanocarrier/detergent (ds*GFP*) or nanocarrier/detergent (negative control) formulations were set as control groups. Number in the column refers to the number of aphids that have chosen the odor sources. Fifteen replicates were conducted for each treatment (*** *P*< 0.001; Student’s t test).


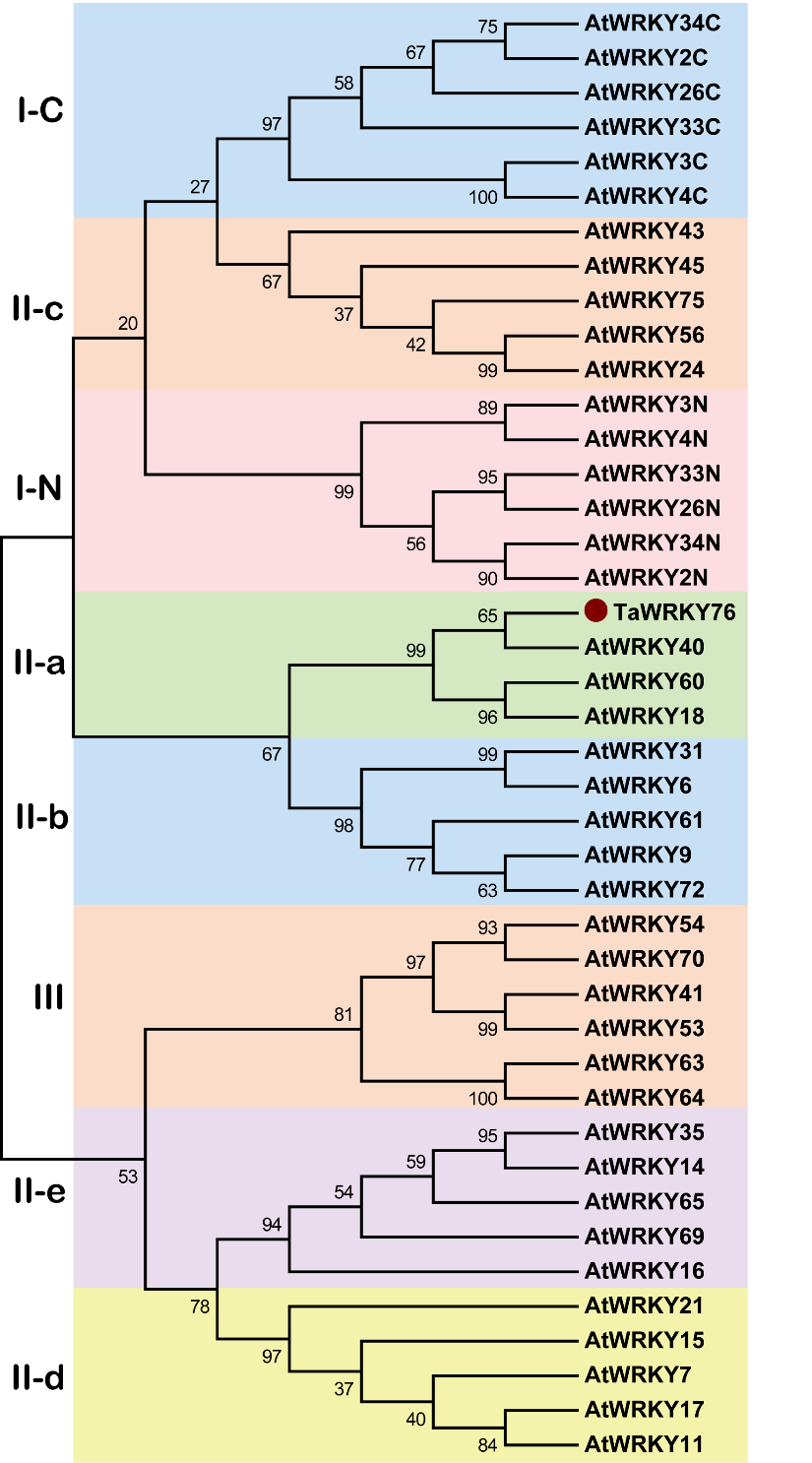


**Figure S5.** Phylogenetic tree constructed by comparing the amino-acid sequences of TaWRKY76 with WRKYs identified from *Arabidopsis thaliana*. Bootstrap values = 1000.


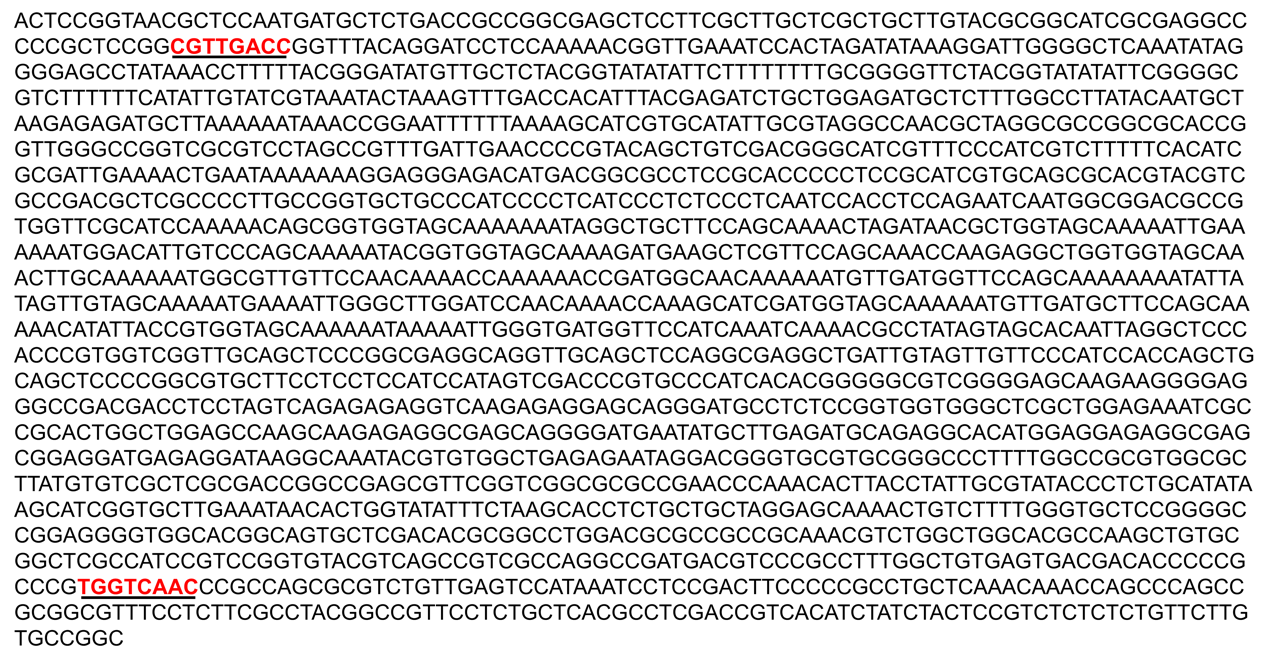


**Figure S6.** The potential binding sites of transcriptional factor TaWRKY76 on the promoter of *DMR6* gene. Letters in red color with underline indicate the predicted binding sites of TaWRKY76 on the promoter of *DMR6* gene. Relative profile score threshold was set as 90%.

**Table S1.** All CSP amino acid sequences used for phylogenetic tree construction.

>AlinCSP1

MLKVLVLLAAVVCCVSAAATYTSKYDNIDLDEILSNTRLYKKYFDCLANKGKCTPDGKELKESLPDALKTNCAKCTKKQQEGTDKVFRHVLKNKPNDYKVLESIYDPPGIYRKKYEAEAEKRGIKLPGSH

>AlinCSP2

MKVAVLVLLCVGAALSAEVYTSKYDNIDVDKILSNDRILTRYIKCLMEEGNCTNEGKELKKTLPDALASGCTKCSEKQKAQTEKVLRHLSKNRPRDWALLKTKYDPKGEYSKKYEKEAKALTA

>AlinCSP3

MISKLSMVLLIGAFADVWAAEQYTDKYDNIDIDEILNNDRMYKNYFHCVMGNGKCTPDGLELKAKIPEALQTECAKCTDKQKKEVEKVLRFIINQKKDDYKLLEEKFDPEGVYRKKYEAQKKLVEEGKPIEY

>AlinCSP4

MRIILSAFLVAMACSLATCEMTEEEFYTKVFEEVDPDFILDNERILTSYLKCFYNEIECNAHAEVVKKSIPDVLATVCGRCSDKQKSIFKYSLNKFIPAHPKDWEKILSIYDPSGEAWPKVKAFIES

>AlinCSP5

MGHLTIVLLAAAFEVLTGSRAYTTHYDYIDVDQVLNNTRLYTKYVECLLGQGKCTPEARELRDKLPEALQTNCARCSERQASESHRVIRFLIQNRQEDFKLLEAKYDPSGLYFKRFEEETKRNVSLS

>AlinCSP6

MFYKLSVVVLMGILAGVWAADKYTDKYDNIDIDEILTNERLYKKYFDCIQGTGKCTPDGIELKEKIPEALKTECAKCNEKQKAGVEKVMRYLITKKPEDFKILEDKFDPEGVYRKKYEAQRKLVEEGKPVEY

>AlinCSP7

MNYKLSVILLIGVLASVWAASTYTDKYDNIDLDEILTNERLYKKYFDCIQGKGKCTPDGTELKEAIPDALKTECAKCNAKQKAGVEKVLRHLLTKKAEDYKILEDKFDPEGVYRKKYEAQKKLADEGKPIVL

>AlinCSP8

MDYKLSVMLLMGVLACAWAADKYTDKYDNIDIDEILNNERLYKKYFDCILGNGKCTPDGTELKETIPDALKTACAKCNDKQKAGVEKVLRHLLTKKAEDYKILEAKFDPEGVYRKKYEAQKKLAEEGKPIAL

>AlinCSP9

LAVVTREMREREFFRQLEVINVDSILINQRLIDKYIKCLLKTGKCDPIMKDLRIALPLILGHLCEARCSEK

>AlinCSP10

MRSNFINESIPDVLATVCGRCSDKQKSIFKYSLNKFIPAHPKDWEKILSIYDPSGEAWPKVKAFIES

>AlinCSP11

MKVFFSGLLLVCMASVSLCADEYTDKYDSVDLDEILNNQRLYQKYIDCVMGKGKCTPDGALLKEKIPEALQNECAKCSAKQKKGAEKVLRFLINEKADDYKALEEKYDPEGTFRSKYEEQKKNLKEGKPLSV

>AlinCSP12

MMIIIVFGISALLVVVEGAPLQYSDTRYDDVELTTILSNDELYIKLFQCLIGRGKCTPDWEILKDALPGALLDNCSECSNKQKFGTKTLLAHLVHERPSDMRLLEGEFDPDGSYRKELEKEEKESNDINRKRSANLEEVEILDKIKRIIK

>AlinCSP13

MKFVAALLVASVAVLAVEAANQYTTKYDNIDLDDILKNQRLYKKYFECLTGNGKCTPDGKELKEHLPDALKTGCSKCSEKQRAGSEKVIKHLLKNKPQDYAVLEKIYDPSGIYKKKYEAEAKKLGINV

>AlinCSP14

MNSAIVLCVVALAGMVLARPDDTYTTKYDNVDLDEILGNDRLLVPYIKCTLDEGKCAPDAKELKEHIREALENGCAKCTDKQKEGTRRVIAHLIKHKNADWQKLKAKYDPEGKYTHKYEKELEEVQH

>AlinCSP15

MKLIVAVALLCVVAESWAASTYTDKWDNINVDEILESQRLLKAYVDCLLDRGRCTPDGKALKETLPDALENECSKCTDKQKSGSDKVIRHLVNKRPEMWKELSAKYDPNNIYQDRYKDKIEAVKGQ

>AlinCSP16

MLPFYVFSLCAVFVACQETYTSKYDNVNVEDALKNDRLYKAYFNCLADRGPCTREGNMLKEALPDGLRNNCSLCTDPQRRGTHQVIRFLFKYRPEDMKLLEEIYDPEGIYKTKYAEERKKLME

>AlinCSP17

MDYKFLVVMQMGVISSVCAAGPYTDKYDNVNLDEVLNNERLYRNYFNCLQGKGKCTLDGAILKEIIPSALKTDCALCSVRQKKGAEKVLIFLITKKPDDFKILEDKF

>AlinCSP18

MIWILLVAVSMTTSLAEEESIDYYRVFEEIDPDLILDNERILQTYLKCFYGEGPCNTHAQLAKESIPDVLA

>AlinCSP19

MVYKSSVVFLLMGTVAYVWGEKYTEQYDDINLDNILTNERLYRIYFKCILSKGKCTPEGEVLKKAIPDALK

>AlinCSP21

KELKEHIKEALENECGKCTEAQKKGTRRVIGHLINHEADFWNELTAKYDPERKYTTKYEKELKEVKA

>AlucCSP1

MLKVLVLLAAVVCCVSAAATYTSKYDNIDLDEILSNTRLYKKYFDCLANKGKCTPDGKELKESLPDALKTNCAKCTKKQQEGTDKVLRHVLKNKPNDYKVLESIYDPTGIYRKKYEIEAEKRGIKLPGSH

>AlucCSP2

MVGKLSVVLLIGAVGMVLAADKYTDKYDNIDVDEILGNQRLYQKYFDCIQGKGKCTPDGAELKKNIPEALQTDCAKCSEKQKAGVEKVLRHLINEKPEDYKVLEEQFDPEGVYRKKYEHLKKKVEEGKPVEY

>AlucCSP3

MLKVLVLLAAVVCCVSAAATYTTKYDNIDLDEILSNQRLYKKYYDCLANKGKCTPDGKELKEALPDALKTNCSKCSKKQQEGTDKVLRYVLKNKPNDYKVLENIYDPSGNYRKRYEDEASKRGIKLPGSH

>AlucCSP4

MVSKLSIVLLIGALADVWASELYTDKYDNIDVDEILGNQRLYQKYFDCIQGKGKCTPDGAELKKNIPEALQTDCAKCSEKQKAGVEKVLRHLINEKPEDYKVLEEQFDPEGVYRKKYEHLKKKVEEGKPIEY

>AlucCSP5

MVGKLSVVLLIGAVGMVLAAELYTDKYDNIDVDEILGNQRLYQKYFDCIQGKGKCTPDGAELKKNIPEALQTDCAKCSEKQKAGVEKVLRHLINEKPEDYKVLEEQFDPEGVYRKKYEHLKKKVEEGKPV

>AlucCSP6

MVSKLSIVLLIGALADVWASELYTDKYDNIDVDEILGNQRLYQKYFDCIQGKGKCTPDGAELKKNIPEALQTDCAKCSEKQKAGVEKVLRHLINEKPEDYKVLEEQFDPEGVYRKKYEHLKKKVEEGKPIEY

>AlucCSP7

MVSKLSIVLLLGALADVWAAELYTDKYDNIDIDEILNNDRMYKNYFNCVMGNGKCTPDGLELKAKIPEALQTECAKCSDKQKKGAEKVLRFIINQKKDDYKLLEEKFDPEGVYRKKYEAQKKLAEEGKPIEY

>AlucCSP8

MLKVLVLLANAASTYTTKYDNIDLDEILSNQRLYKKYYDCLANKGKCTPDGKELKEALPDALKTNCSKCSKKQQEGTDKVLRYVLKNKPNDYKVLENIYDPSGNYRKRYEDEASKRGIKLPGSH

>AsutCSP1

MLPFYVFSLCAVFVACQETYTSKYDNVNVEDALKNDRLYKAYFNCLADRGPCTREGNMLKEALPDGLRNNCSLCTDPQRRGTHQVIRFLFKYRPEDMKLLEEIYDPEGIYKTKYAEERKKLME

>AsutCSP2

MGHFPPVFSLSPVLLVASLHTMNTSTLLKIAFLLGCVAACLAAETRSSVSDEALEAALKDKRYLTRQLKCALGEGACDPVGRRLKTYAPLVLRGACPKCTPSEVRQIQQVLSHIQRHYPKEWAKILKQYAGQ

>AsutCSP3

MKFVAALLVASVAVLAVEAANQYTTKYDNIDLDDILKNQRLYKKYFECLTGKGKCTPDGKELKEHLPDALKTGCSKCSEKQRAGSEKVIKHLLKNKPQDYAVLEKIYDPSGIYKKKYEAEAKKLGINV

>AsutCSP4

MRIILSAFLVAMACSLATCEMTEEEFYTKVFEEVDPDFILDNERILTSYLKCFYSEIECNAHAEVVKKSIPDVLATVCGRCSDKQKSIFKYSLNKFIPAHPKDWEKILSIYDPSGEAWPKVKAFIES

>AsutCSP5

MDYKFFVVMQIGVISSVCAAGTYTDKYDNVNLDEVLNNERLYRNYFNCLQGKGKCTLDGAILKEVIPSALKTDCALCSVRQKKGAEKVLIFLITKKPDDFKILEDKFDPEGVYRKKYEAQRKLVEEGKPIH

>AsutCSP6

MVCKLFAVVLMGILAGVWAADKYTDKYDNIDIDEILTNERLYKKYFDCIQGIGKCTPDGIELKEKIPEALKTECAKCNEKQKAGVEKVMRYLITKKPEDFKILEDKFDPEGVYRKKYEAQRKLVEEGKPVEY

>AsutCSP7

MVSKLSMVLLIGALADVWASELYTDKYDSIDIDEILNNDRMYKNYFNCVMGNGKCTPDGTELKAKIPEALQTECAKCSDKQKKGVEKVLRFLIKEKKDDYKLLEEKFDPEGVYRKKYEAQKKLVEEGKPIEY

>AsutCSP8

MDYKLSVMLVMGVLACAWAADMYTDQYDNIDIEEILTNERLYKKYFDCIIGNGKCTPDGTELKETIPDALKTACAKCNDKQKAGVEKVLRHLLTKKAEDYKILEAKFDPEGVYRKKYEAQKKLAEEGKPIVL

>NlugCSP1

MFKNVLLVCLLVAVVSAKPKPAEKKQYTTKYDNIDLDEILNNQRLFDNYYKCLLGGKCTPDGQELREALPDALATACSKCTEKQRVGTEKVIKYLIEKKPTEYSELEKKYDPQGNYKRKYQAEAAKRGIKV

>NlugCSP2

MSKLPVTLVLMLAVFSVDCGKLYKDRYTTKFDKIDLDEALNNQRLFESYLKCLMGDKCSPDGYELREALPDALATACAKCSEAQKAGTEKVIRFLIEKRPKEYALLEKKYDPEGIYRDKYKPIAEMKGIKLD

>NlugCSP3

MKFLCVTIFECALIVVAFGMPQDTTYPTTYDDVNVDDILHNDRLFNRYFTCLTKKEGCTPEGKLLAATIPDALATTCAKCSAKQKTAAEKVIKYLYFNKRDKFDELAKIYDPESNYLNKYLVDGFPAKV

>NlugCSP4

MFLIAVWALSPRRLPWGLPWGGLAGVAAQQQAKNTRYTTRFDSIDVEVILKNERIFRRYMDCLLDKGRCTPEARELKRLLPEALKTECLKCSEVQRRQGAKVMAFIIKNKRPSWELLLAKYDPQGIFRAKYMYNENNIEAVLKQLEREQQGIYGTYSSTNSTTSSNSTSIR

>NlugCSP5

MRCLLLVAVVCAALVAVCHAQDSKYTSKYDNIDIDKILKNDRVLSQYIKCLMGEGSCTQEGRELKRLLPDAIQSNCSKCSEKQRSASVKVMRHLRQSRERDWNRLLDKYDPQGDKRKNLKLD

>NlugCSP6

MLWAARFIVLPLLFCVLQVWSAPADEKYTDIDFDSILANRRVLSSYVKCLTDKGPCTPQGKELKKIVPEVIQTSCTKCSPQQKKVVRNVITTMQSKYKDQWDLVVNKYDPKKQRSGELKAFLSGTD

>NlugCSP7

MIQSTQTFISIALLLFIQIAILSMASASSGTTSTTSAPKTAESASAKSSSKDEIPDQTFDRYINNERYMLMQYECLMGNKPCDHVGRKLKAAVPLVVRGLGCPKCSQREEDQMKRIVSHVQRSYPDKWQKLIKKYGN

>NlugCSP8

MSSTMLVFVAVLCFSAVLAKPADKYTTKYDNIDLDEVLSNQRLFDSYFKCLMGGKCTPDGQELRDALPDALATACEKCSEKQKEGTEKVMKFLIEKKPTEFAELEKKYDPQGTYRQKYKAEADKRGYSV

>NlugCSP9

MKSQQLLVSCLFICTWLVVLMAPSANAAPKEKDPERKALYRLEYIDIEKVLDNNRMLTNFIRCFLRQGPCTPEARDFRKLLPKLAKTMCSDCTARQRYIIKKVFKHLMEERPKEWELLMDRFDPQRKYAERLDTFMVDMTTRAPVTSSPMPSSPVTLTSSSVTMSSTTQRVIEILRTSTDMSNESRPAS

>NlugCSP10

MFMLLACSELGSGQQQQNVDNIEMSIYDKMFENMDVNSLLKNHRLVDSYLKCFLNEGSCTHIGHEVKMMIPEVIKSRCGTCGENQMRALKAGLRLFIVLRPDDWQRFLDVYDPDRKEWPHIKAFMDSDD

>NlugCSP11

MKSIILLVFVSMSAMVYRCRADEPSYPTSWDNVNIDEVLGNERLVQNYAKCLLEKGSCSPEGTELKKAIPDALKTGCTKCSDKQKAGAQKVIKWLVQKKPELWKEVVDKYDPSGEYTKKYEKEYQI

>SfurCSP1

MFNLLTLVVCLSTIAVQIQAAPEEAQYTTKYDKINLDEILNNDRLFKSYFGCLMGGKCTPDGQTLRDILPDALETACSKCSDTQKAGTEKVFKFMIEKKPSEFADLEKKYDPNGKYRARYEADAEKFGIKV

>SfurCSP2

MVLADTPTTSPKVETKAVESGKSSSKDEIPDQTFDRYINNERYMLMQYECLMGNKPCDHVGRKLKAAVPLVVRGLGCPKCSQREEDQMKRIVSHVQRSYPDKWQKLIKKYGN

>SfurCSP3

MQLLYALVFGCTLVMVSSDMPQSTYPTKYDDYNPDDILKNDRLFNQYFICLTKKKGCTTAGELLSAIIPDALATSCAKCSAKQKAIGEKVIRFLYFNKPDEFAEMSKIYDPEGKYLEMYIASGGLI

>SfurCSP4

MKCPLLSVSCLWISLLALSSSASAATKEKDPERKALYRLEYIDIEKVLDNNRMLTNFIRCFLRKGPCSPEARDFRKLLPKLAKTMCSDCSPRQRFIIKKVFKHLMEERPKEWELLMDRFDPQRKYAERLDTFMVDMTTPSTTTTTTSTTPSTPMSSTTQRIIEILRTSTEMSNESSP

>SfurCSP5

MSEILVTSLIFMLLAASELGLGQQQQTQKPQQQNVDNIEMSIYDKMFENMDVNSLLKNHRLVDSYLKCFLNEGSCTHIGHEVKMMIPEVIRSRCATCGENQMRALKAGLRLFIVRRPDDWKRFLDVYDPDRTEWPHIKAFMESDD

>SfurCSP6

MKLALFCCLLGLVAAVSAQSEKSEKPEKYTTKYDYINVDEILSNDRLFNSYYKCLMGGKCTPGGPELRTHLPDALQTNCSKCSEKQKEFSDKVIKHLMDNKPEEFSALVKKYDPEGIYKDAFKPKHNQ

>SfurCSP7

MRASKASSLVAVLLIAVWGFTGVQAQQKSKDTRYTTRFDSIDVEVILKNERIFKRYMDCLLDKGRCTPEARELKRLLPEALKTECLKCSEVQRRQGAKVMGFIIKNKRPYWDLLLAKYDPQGIFRAKYNYNENNIEGVLKQLEREQQGLYGTYSNTTNTTNTVNSTSTRK

>SfurCSP8

MLKFTLTLLVLAVVSVNCGKLYKDRYTTKFDKIDLDEALNNQRLFESYLKCLMGDKCSPDGYELREALPDALATACAKCSDAQKAGTEKVIRFLIEKRPKEYALLEKEYDPEGIYRDKYKPIAQEKGIKI

>SfurCSP9

MRCLLLVAVVFAAFIAAARADEANKYTSKYDNIDIDKILKNDRVLSQYIKCLMGEGSCTQEGRELKRLLPDAIQSNCSKCSEKQRQASVKVMRHLRQSKERDWNRLLDKYDPQGDKRKNLKLD

>LstrCSP1

MASVSSATLTAAALLALLALQLTAAQNFNEADIARMLNDSGLVQRQISCILGEAACDNIGNMLKLAIPEVLKRNCRSCNAQQASNARRLISFVQANYPAQWQRIQSRYVG

>LstrCSP2

MRCLLLVAVICAAFIAAAQADEANKYTSKYDNIDIDKILKNDRVLSQYIKCLMGEGSCTQEGRELKRLLPDAIQSNCSKCSEKQRQASVKVMRHLRQSRVRDWNRLLDKYDPQGDKRKNLKLD

>LstrCSP3

MKLALFCCLLGLVIAVSAEKYTTKYDHINVEEILNNERLFNSYYKCLMGGKCTPDGLELRTHLPDALRTNCSKCSEKQKEFSDKVIRYLIDNKPEEFAALTKKYDPEGIYKTTFGPQFKKDNTTTNQ

>LstrCSP4

MLWAAKFIVFPLIFCVLQVWSAPADEKYSDIDFESILANRRVLSSYVKCLTDKGPCTPQGKELKKIVPEVIQTSCTKCSPQQKKVVRNVITTMQSKYKDQWDLVVNKYDPKKQRAGELKAFLAGTD

>LstrCSP5

MLKFKLTLLVMASAFFSVDGGKLYKDRYTTKFDKIDLDEALNNQRLFESYLKCLMGDKCSPDGYELREALPDALATACAKCSEAQKAGTEKVIRFLIEKRPKEYALLEKKYDPEGVYRDKYKPIAEEKGIKI

>LstrCSP6

MQASSLAMLLIAVWVLSPRRPLSGGFAGVHAQQSKNTRYTTRFDSIDVEVILKNERIFKRYMDCLLDKGRCTPEARELKRLLPEALKTECLKCSEVQRRQGAKVMAFIIKNKRPYWDLLLAKYDPQGVFRAKYKYNDQNIEAVLKQLEREQQGLYGTYSNPTNTTTVNSASSRK

>LstrCSP7

MIQRTQGFNSIVVLLLIKLTVLSMVLASTHAPAPTPTPKVETKATEAAKSSSKDEIPDQTFDRYINNERYMLQQYECLMGNKPCDHVGRKLKAAVPLVVRGLGCPKCSPREEEQMKRIVSHVQRSYPDKWQKLIRKYGQ

>LstrCSP8

MKSPCLLSVSCCILVLVASSASAAPKEKDPERKALYRLEYIDIEKVLDNNRMLTNFIRCFLRKGPCSPEARDFRKLLPKLAKTMCSDCSPRQRFIIKKVFKHLMEERPKEWELLMDRFDPQRKYAERLDTFMVDMTTRATPTTTTTTIPTTTTPMSSTTQRIIEILRTSTEMSNESRP

>LstrCSP9

MSEVLVMILIFMLLAGREQRLQQQQQQQQQPQPQQQNVDNIEMSIYDKMFENMDVNSLLKNHRLVDSYLKCFLNEGSCTHIGHEVKMMIPEVIRSKCATCGENQMRALKAGLRLFIALRPDDWKRFLDVYDPDRTEWPHIKAFMEYDD

>LstrCSP10

MYDHINVYNILKNERLFNRYFTCLTKKEGCTPEGKLLAAAILDALETSCANCSNEQRKLAEQVIQYLYFNKRDKFDELAMIYDLEGVFQEYHIAEYLVSGSWMPDFRKLPPV

>LstrCSP11

MKFLYFTVFGCALVMFTSAIPEIIFTSAMPQKTYSTMYDHINVNNILKNDRLFNRYFTCLTKRGGCTPEGKLLAAAILDALETSCANCSNEQRKLARQVIQYL

>LstrCSP12

MFKNLLVVCLLVAAVSAKPKPAEKKNTTKYDNIDLDEILNNQRLFDNYYKCLLGAKCTPDGQELKEALPDALATACSKCTEKQRVGTEKVIRHLIEKKPTEYAELEKKYDPQGTYKRKYQAEAIKRGIKV

>SmisCSP2

FVVLVASLVCFTLAEEKYSTKYENFDVDKVLNDDSLLTSYINCLLDEENCTEEGQALKRVLPDALKTNCGKCTDTQKMKIEKILKFLMKNRSTDFDRLTAKYDPSGEYKKKLEKFSA

>SmisCSP4

MDSRIAVVCVVLAVFAVDQTVGAPQKDALAAGSPTTYTNKYDHIDIDQVLASKRLVNSYVQCLLDKKPCTPEGAELRKILPDALKTQCAKCSATQKNAALKVVDRLQKDYDKEWKQLLDKWDPKREQFQKFQQFLTEEKKKGVVKF

>SmisCSP5

MNCKVLIALCCVAVYAAQANPAGAATATAADDEIKDFPAYMKRFDKLNVEQVLNNDRVLASHLKCFLNEGPCVQQSRDLKRVIPVIANNGCNGCTERQMTTIKKSLNFLRTKKPVEWARLVKIYDPSGTKLNKFLDA

>SmisCSP7

MARSSSTSVTMKVFVMAVCVCAALARPEEAKMENKPTAVKSETLAAPLPTTIVKRATPQVVSIQKDASLPNVSEDVLDKALSDRRFVQRQLKCATGEGPCDPIGRKIKAHAPLVLRGMCVKCSQSEIKQIQRVMSHIQKNYPKEYTKMLKQYQSGF

>SmisCSP1

MNLLAIFCYITMMCDSQFRRLEQPTAIPQVKRIEQPATIATRIGQATIAPRFGQPTVAPRFGQPTIAPRFGQATAAPQTGEAAIGPRIGQTFQNVNDSVSPTTDGRKTTRETSSYPTRYDFIDIEAVMNNDRIIKILFNCVMNQGPCTREGLELKRIVPDAIQTECAKCNERQRKQAGKVLAHLLQYKPEYWNMLVKKFDPNNIYLRKYMADNDDDEKLSLQKLSNNTTK

>AgosCSP1

MNILTIFCYVTVMCDTQVKPAVSAQRLQSVNQNVTPTNDGRKTIRETSSYPTRYDYIDIEAVMNNERIIKILFNCVMSRGPCTREGLELKRIVPDAIQTECAKCNERQRKQAGKVLAHLLQYKPEYWKMLVQKFDPNNVYLRKYMADNDDDEKLSLQKLSNDTTKKKRNI

>AgosCSP2

MAHLNLFVVLIASLIYFTSAAEEKYTTKFDNFDVDKVLNNNRILTSYIKCLLDEGNCTNEGRELKRVLPDALKTDCSKCTDVQKDRSEKVIKFLIKNRSTDFDRLTAKYDPTGEYKKNLEKFEKERASAKPLKA

>AgosCSP4

MDSRIAVVCVVLAAFAVDQTVGAPQKDAVAASGPAYTTKYDHIDVDQVLASKRLVNSYVQCLLDKKPCTPEGAELRKILPDALKTQCAKCNATQKNAALKVVDRLQKDYDAEWKQLLDKWDPKREHFQKFQQFLAEEKKKGFTKF

>AgosCSP5

MHCKVLIALCCVAVYAVQASPAGTATAAAVSADDEIKDFPAYMKRFDKLNVEQVLNNDRVLASHLKCFLNEGPCVQQSRDLKRVIPVIANNGCNGCTERQMTTIKKSLNFLRTKKPTEWARLVKIYDPSGTKLNKFLDA

>AgosCSP6

MIKLILAIAFCVSITMTVVQTAPAKYTTKYDNVNIDEILNNDRLVASYFKCLMETGKCTPEGEEIKRWLPEAIENKCEDCSEKQKLGSEKIIKFLFEKKNDMWKQLEAKYDPQGTYRQRYAEEAKKLNINV

>AgosCSP7

MSRSSSSVTMKVFVIAICVCAALARPEDVKVENKPAVIKSETLAAPLPTNIVKRATDTIQLDSSLPNVSEDVLDKALSDRRFVQRQLKCATGEGPCDPIGRKIKAHAPLVLRGMCVKCSQSEIKQIQRVMSHIQKNYPKEYTKMLKQYQSGF

>AgosCSP8

MNNIIMNNSRGRYGIFSLLAVTIAAIMLVHQPATVRCADGGIITPQQQQQQTMMFTAPTGYYVSTYDHIDVGRLLRNNKVVSGYVKCFVNEGPCTPDGKLVKAYLLPEIIRTVCGKCTPRQKDMARMVLKHIYTYRQADFEKIMQIYDTDGKRNEILAFMNH

>AgosCSP9

MSAFCLNSFILMTMITVIVTHATFTRSTKFDDRTGIDIHLVKRDTDDVNDDENSVESDEGFFYRFTHFFQDSSDKEDDDDDEKKPDFITTFDIFKLLDEEYAMQQFYCVINEDPCDEVGMRLKATIPEEINRNCERCTSTERNNIRRILNYVKKHYPQFWKRVEPIYKKKI

>AgosCSP10

MINTRPRKLVRCIRGVSISVAKGDDAVNAENKDDDSHLVNREEIQRYMSMMEKINIDQMLNNTRLMSNNVKCFLNEGPCTAHLREMKKMVPMLVKDSCSSCTKEQKIMMKKAMDAVKARRPNDYEKLSKFFDPEGKYEKKFLENLNESK

>MperCSP1

MNLLAVFCYITMMCDSQLFKRLEQPAAISQVKRIEQPAMIANRIGQPTVAPRFGQPTIAPRFGLPTIAPQVGQAAITPQVGQAAIASRFGLPTVAPQVGQAAITPQVGQAAIASRFGLPTVAPQVGQAATTPQVGQAAIASRIGQNFQNANNSVSPTTDGRKTTRETSSYPTRYDFIDIEAVMNNERIIKILFNCVMNQGPCTREGLELKRIVPDAIQTECAKCNERQRKQAGKVLAHLLQYKPEYWNMLVKKFDPNNVYLKKYMADNDDDEKVSLQKLTNDTTK

>MperCSP2

MAHLNLFVVLVASLVCFTLAEEKYTTKFDNFDVDKVLNNNRILTSYIKCLLDEGNCTNEGRELRKVLPDALKTDCSKCTEVQKDRSEKVIKFLIKNRSTDFDRLTAKYDPSGEYKKKIEKFDSEKAAAAKH

>MperCSP4

MDSRIAVVCVVLAVFAVDQTVGAPQKDAVAASGPAYTTKYDHIDIDQVLGSKRLVNSYVQCLLDKKPCTPEGAELRKILPDALKTQCVKCNATQKNAALKVVDRLQRDYDKEWKQLLDKWDPKREYFQKFQQFLAEEKKKGVVKF

>MperCSP5

MNCKVLIALCCVAVYAAHASPAGAATAAAASADEEIKDFPAYMKRFDKLNVEQVLNNDRVLASHLKCFLNEGPCVQQSRDLKRVIPVIANNGCNGCTERQMTTIKKSLNFLRTKKPVEWARLVKIYDPSGTKLNKFLDA

>MperCSP6

MNTLLLAVALCIAITMTVVQTAPAKYTTKYDNVNIDDILNNDRLVASYFKCLMETGKCTPEGEEIKRWLPEAIENKCENCSEKQKIGSEKIIKFLIEKKNDMWKQLEQKYDPQGLYKQRYSEEAKKLNLDV

>MperCSP7

MDRSSSSVTMKVFVIAVCVCAALARPEDSKVENKPAAVKSETLAAPLPTTIVKRATPQVVSTQQGASLPNVSEDVLDKALSDRRFVLRQLKCATGEGPCDPIGRKIKAHAPLVLRGMCVKCSQSEIKQIQRVMSHIQKNYPKEYTMMLKQYQSGF

>MperCSP8

MTNNNMNSPRCRPEIFSLLAVAAIATVLVHQPSTVHCADAGVYPPQQQQQEATMFTAPSGYYVSTYDHMDVGRLLRNNKVVAGFVKCFTNEGPCTPEGRLAKAYLLPEIIRTVCGKCTPRQKDMARLVIRHIYTYRRGDFDKIMQIYDTDGKKNEIIDFMNQK

>MperCSP9

MTSFCLNSVILMTITTVIVAHAASTGMTAFNNRSGSDIHMAQRDYNENKADKAEGFFFTITNFFSRRKHDDDKPDFITTFDIIRLLDEKYAMKQFYCVINKEPCDATGLRLKATIPEEINNDCERCTATETSNIRRILNYVKKHYPEFWDRVEPIYRNNMTA

>MperCSP10

MVSKLFVSVFVLMSVVGVSYSVTEGDDDAAKVADKDLHPVNQEELKKFLSMMEKVDIDQILNNNRLMSNNVKCFLNEGPCTGQLREMKKMVPMLVKDSCSSCNKEQKNMMKKAMDAMKARRPNEYEQISKFFDPEGKYEKKFLENLNESK

>ApisCSP1

MNLLAIFCYITMMCDSQFRRLEQMTAMPQVKQPATIATRIGQATIAPRFGQPTIAPRFGQATVAPQVGQAAVTPQIGQAAIGSRIGQSFQSVNGSVTPTTDGRKTTRETASYPTRYDFIDIEAVMNNDRIIKILFNCVMNQGPCTREGLELKRIVPDAIQTECAKCNERQRKQAGKVLAHLLQYKPEYWNMLVKKFDPNNIYLRKYMADNDDDEKLSLQKLTNNTTK

>ApisCSP2

MAHLNLFVVLVASLVCFTLAEEKYTTKFDNFDVEKVLNNDRILTSYIKCLLDQGNCTNEGRELKRVLPDALKTDCSKCTDVQKDRSERVIKFLIKNRSAEFDKLTAKYDPSGEYKKKIEKFDAERAAAAKH

>ApisCSP3

MVHLNLFVVLVASLVCFTLAEEKYTTKFDNFDVEKVLNNDRILTSYIECLLDQGNCTNEGRELKRVLPDALKTDCSKCTDVQKDRSERVIKFLIKNRSAEFDKLTAKYDPSGEYKKKLEKFSA

>ApisCSP4

MDSRIALVCVVLAVFAVDQTVGAPQKDAASGPVYTTKYDNIDIDQILASKRLVNNYVQCLLDKKPCTPEGAELRKILPDALKTQCSKCNPGQKNAALKVVDRLQKDYDKEWKLLLDKWDPKREQFQKFQQFLVEEKKKGVVKF

>ApisCSP5

MNCKILIALCCVAVYAAQANPAGVATATAADEEIKDLPAYMKRFEKLNVEQVLNNDRVLASHLKCFLNEGPCVQQSRDLKRVIPVIANNSCNGCTERQITTIKKSLNFLRTKKPVEWARLVKIYDPSGVKLNKFLDA

>ApisCSP6

MNKLFLAVAFCIVTMMTVVQTAPAKYTTKYDNVNIDDILNNDRLVNSYFKCLMETGKCTPEGEEIKRWLPEAIENKCEDCSEKQKLGSEKIIKFLIEKKNDMWKQLEEKYDSKGLYRQRYSEDAKKLDIHI

>ApisCSP7

MARSSSSVTMKVFVIAVCVCAALARPEEAKMENKPAVVKSETLAAPLPTTIVKRATPYVVSTQQDSSLPNVSEDVLDKALSDRRFVQRQLKCATGEGPCDPIGRKIKAHAPLVMRGMCVKCSQSEIKQIQRVMSHIQKNYPKEYTKMLKQYQSGF

>ApisCSP8

MTNNNMNCPRSRPEIFSLLTVTAIAAVLVHQPTTVYCADGGTYPQQQLQQQQQQQQQQQQQQQFTAPSGYYVSTYDHIDVGRLLRNQKVVSGYVKCFVNEGPCTPDGKLVKAYLLPEIIRTVCGKCTPRQKEMARMVLRHIYTYRRADFDKIMQIYDTDGKKNEIINFMNQK

>ApisCSP9

MSSFCLNSVILMTVITVVVARVAFAESTTSNDRPGSDIRLVKKDVDYNEDDADDREEGFFFRISHFFGFTSYDDDKPDFITTFDLIRLLDEKYAMKQFYCVINEEPCDAVGLRLKATIPEEINRDCERCTATETSNIRRILNYVKKHYPKFWERVEPIYRNNTTA

>ApisCSP10

MVSKRFISVFMFMAVVGVSFSVPEDDDATKVVNKEVDHHSVIQEEIKKFLSMMEKINIDQILNNDRLMSNNVKCFLNEGSCTAQLREMKKMLPVLIKDSCSSCTKEQRNMIKKAMDAIKARRPNEYERVTKFFDPEKKYEKKLSEKLNES

>AglyCSP1

MNILTIFCYVTVMCDTQVKPAVSAQRLQSVNQNVTPTNDGRKTIRETSSYPTRYDYIDIEAVMNNERIIKILFNCVMSRGPCTREGLELKRIVPDAIQTECAKCNERQRKQAGKVLAHLLQYKPEYWKMLVQKFDPNNVYLRKYMADNDDDEKLSLQKLSNDTTKKKRNI

>AglyCSP2

MGINIYKLKRIKMAHLNLFVVLIASLIYFTSAAEEKYTTKFDNFDVDKVLNNNRILTSYIKCLLDEGNCTNEGRELKRVLPDALKTDCSKCTDVQKDRSEKVIKFLIKNRSTDFDRLTAKYDPTGEYKKNLEKFETERATAKPLKA

>AglyCSP4

MDSRIAVVCVVLAAFAVDQTVGAPQKDAVAASGPAYTTKYDHIDVDQVLASKRLVNSYVQCLLDKKPCTPEGAELRKILPDALKTQCAKCNTTQKNAALKVVDRLQKDYDAEWKQLLDKWDPKREHFQKFQQFLAEEKKKGFTKF

>AglyCSP5

MHCKVLIALCCVAVYAVQASPAGTATAAAVSADDEIKDFPAYMKRFDKLNVEQVLNNDRVLASHLKCFLNEGPCVQQSRDLKRVIPVIANNGCNGCTERQMTTIKKSLNFLRTKKPTEWARLVKIYDPSGTKLNKFLDA

>AglyCSP6

MIKLILAIAFCVTITMTVVQTAPAKYTTKYDNVNIDEILNNDRLVASYFKCLMETGKCTPEGEEIKRWLPEAVENKCEDCSEKQKLGSEKIIKFLFEKKNDMWKQLEAKYDPQGIYRQRYAEEAKKLNINV

>AglyCSP7

MYMGNPSPSIDRIWSHYCHHLNTRSMSRSSSSVTMKVFVIAICVCAALARPEDVKVENKPAVIKSETLAVPLPTNIVKRATDTIQLDSSLPNVSEDVLDKALSDRRFVQRQLKCATGEGPCDPIGRKIKDILGADPSSRTASVERNVRQVFTVGNQTDSTCHVPYSEELSQGVHQDAETVPERILITMRRPCTIFLFMTSGFWHNLY

>AglyCSP8

MNNSRGRYEIFSLLAVTIAAIMLVHQPATVRCADDGIITPQQQQQQTMMFTAPTGYYVSTYDHIDVGRLLRNNKVVSGYVKCFVNEGPCTPDGKLVKAYLLPEIIRTVCGKCTPRQKDMARMVLKHIYTYRQADFEKIMQIYDTDGKRNEILAFMNH

>AglyCSP9

MSAFCLNSFILMTMITVIVTHATFIRSIKFDDRTGIDIHLVKRDTDDVKDDENSVESDEGFFYKITHFFQHHDKEDDDDDEEKPDFITTFDILKLLDEEYAMEQFYCVINEDPCDEVGMRLKATIPEEINRNCERCTSTERNNIRRILNYVKKHYPQFWKRVEPIYKKKI

>AglyCSP10

MNSKIFISVFMFITIVSVSISVAERDDAVKAENKDDDSHPINREEIQRYMSMMEKINIDQMLNNTRLMSNNVKCFLNEGPCTAHLREMKKMVPMLVKDSCSSCTKEQKIMMKKAMDAVKARRPNDYEKLSKFFDPEGKYEKKFLENLNESK

>BtabBCSP1

MHLFSVVVLVCCLLVAVLSAPAEFYTSQFDNIDIESILKNEKLLDNYFNCLMDEGPCTLEGRTLKSLLPDALNTSCAKCTEKQKKIARRVMTFYLDKYPANSARIIKKYDPENKFKDGIEKALLGSR

>BtabBCSP2

MFKVLVVLCVLGAAFVYAAPAEDKYTDKYDNINVDDILGSKRLLKSYLTCLLDKSPCTPEGSELKRLLPDALKTACSKCTEKQKEGAARIVERVTAEYPTEWKELSAKWDPTGEYWAKYKPLVQEYLKASA

>BtabBCSP3

MQVLTLVVLVGCAATAVLSADTYTTQFDNIDLEAILKNEKLVDNYTKCLMDEGPCTNEGRTLKKLLPDALKTACAKCTEKQKTGARKVIKFYQTQHPEDFKKLQQKYDPEGKFKAEFEKALFGQTL

>BtabBCSP4

MRCACVLLVLVICVWGTSGQRVGEGDVSRLLTNRDYVNRQINCVLDKGSCDNIGRQLKQAIPEVLGRQCKSCSARQLDNARKVVNYIRSNYPGPWSQIEAKYGRAAF

>BtabBCSP5

MKSVCVFAALVVACYAAPPAGVDEKLLSKYDNFDVDRVLNNDRVLANYIKCLMDEGSCTNEGRDLKKSIPDVLAGGCDKCTEKQKMVTEKVIKHLINKRPKDWDRLSKKYDPQGQYKNKYADLYEKVQKEAAKESKEPSKPTKDTTKVTKDTKESAKAPKA

>BtabBCSP6

MNKIVLALFALCALFGFSSAAAATKESTKESTYTNKYDNIDLGKILTNDRLFLNYFKCLMDEHTCSPDGAELKKVLPDALSNKCAKCTERQRSGSEKVIRHLIDNKPEMWAKLEAKYDPKGTYRKTYKNEAEKLGIKV

>BtabBCSP7

MIRVTLLLVAVALVGFVAGAPAPLEQSDLEKFENMDLSSILSNKRLRTAYVNCMVDKGPCTADAAEFKKILPDLTETQCADCSAKFKELIKKSVSTFQKDYPEDWKTLMAHFDPDNKRAADLEKFMSS

>BtabBCSP8

MKCVVLLAIFSVLAVYFVESAHLGPNFGAGDISGHLKNKNYILKQLNCVLGKGACDNVGKQLKVAIPEVLNKNCKGCTSQQAANAKRLITFMKSNYPAEWSKIAAKYKK

>BtabBCSP9

MSKYVFVLCVVAALAAVVSAADDFYSDKYDNIDLDSILASKRLIRNYMNCFQGKSPCTPEGTYLNQVLPEALKTECAKCTEKQREGAVKAIKKLSAEYPEEWKEITDKLDPTGEQYAKFKARFP

>BtabBCSP10

MGTFRALLLVVVSVCVFNVLRATPVPDEEKYSDKYDDVDYKSILNSKRLLNNYVKCLLDEGPCTAEGKALRDQLPDILATECKKCTDKQKKGSLDILEILQTEHQDAWTVLAKRWDPEDKLTKPLMEKLKKETGQA

>BtabBCSP11

MVCSESIVRCLTYFQVISLVYRLASAQSNNRATYTTKYDYINVDAVMKNERILKMLVECMLERGRCTREGLELKAAVPDALATDCAKCSQMQRKHASRVIAYLITYKKEYWNALATKYDPDGSYRRKYGIPPQPQELSAGAAIQPSPNLIKPTKTVKKTVTTVNNTNNLFKAKKRMTREEKKEMVRRRFPQLHFMPNMWASNIQKIEKKSERAPSIARRNVRRKAHTPTRRHSTQRTAPKRGQETG

>BtabBCSP12

MNPTVFLVILGQLSFVFSAISDDEYRLETLCSSPALEHFDITPILKNDRLVSSYFKCFMDEGPCTNEGKMVKRIIPEIMRTQCRNCNPTMRRIVRTVMKHMFQTRPRDVDDFFLKYDPHEMYYDDLIEFMDEDNDY

>BtabBCSP13

MNSLVLCAFVGSFIVGTLAAPAETYTTEFDGIDIDSVLKNEKLLDAYAKCLLDEGPCTREGRTLKTLLPDALETTCAKCSPTQKEKAKKVITFYMEKYPENAQKIMKKYDPTGKYRKALEEAFLGSL

>BtabBCSP14

MMKYCALSAVLACVFVVAVGRAQENQKQVQVPVNEMLNNTRMREAYFKCMSDKGPCTPDAAELKKVLPEAMTKKCAACTDTQKKILSKILDYMMEKDKATYKEIQEKYDPKNEYTKMREEEIKKEKAEEKKKPAEKDAPKKS

>BtabBCSP15

MIYVQILSFLCLSVLLAEAMPAPQTTRATISDEALESALNDKRYLMRQLKCALGEGVCDPVGRRLKTFAPLVLRGACPQCSPTETRQIQKVLSHIQRHHPKEWSKIVKQFTS

>BtabBCSP16

MFRLLLVTSLVLLVTGLPQKGPASTPRKQSVEEALGKKPEELPKTMKEALKRMEAVDVEKVLNNDRILTNYLKCFLNKGPCTSEAKNVKKFIALLVESRCVECDPKQRKIIKKSMQVVKTKKPREYQELIKLYDPKGTQIAELEKFFASSK

>BtabBCSP17

MNFLSVVVLVCCLFAAVLSAPAEFYTSQFDNIDIESILNNEKLLDNYFKCLMDEGPCTLEGRTLKSLVPDALNTSCAKCTDKQKQIARRVITFYLDKYPANSARIIKKYDPENKFKDGIEKALLGSR

>BtabBCSP18

MNLLSVVVLVCCLFAAVLSAPAEFYTSQFDNIDIESILNNEKLLDNYFKCLMDEGPCTLEGRTLKSLVPDALNTSCAKCTDKQKQIARRVITFYLDKYPANSARIIKKYDPENKFKDGIEKALLGSR

>BtabBCSP19

MHLLSVVVMVCCLVAAVLAAPAEFYTSQFDNIDIESILRNEKLLDNYFKCLMDEGPCTSEGRFLKNLLPDALNTKCAKCTDKQKKIARRVMTFYFDKYPANAARAIKKYDPENKLKDGLEKALLGAR

**Table S2.** All primers used in this study.

| Primer name | Primer sequence (5’-3’) | Purpose |
| --- | --- | --- |
| *SmCSP4*-qPCR | F: GACAAGAAACCGTGCACACC  R: GGGTCCCATTTGTCAAGAAGC | qPCR for aphids |
| *NADH dehydrogenase* | F: CGAGGAGAACATGCTCTTAGAC  R: GATAGCTTGGGCTGGACATATAG |  |
| *β-Actin* | F: CGGTTCAAAAACCCAAACCAG  R: TGGTGATGATTCCCGTGTTC |  |
| pCAMBIA  1300-*SmCSP4* | F: GGGGTACCATGGCGCCGCAAAAAGATG  R: CCTCGAGAAATTTAACAACCCCTT | Subcellular localization |
| pCAMBIA  1300-*TaWRKY76* | F: GGGGTACCATGTGTTGCTTCTGGACCAT  R: CCTCGAGCTAATAATCCGGCAGCTTC |  |
| *SmCSP4*-T7 | F: TAATACGACTCACTATAGGG  ATGGATTCACGAATTGCAG  R: TAATACGACTCACTATAGGG  TTAAAATTTAACAACCCC | RNAi |
| *GFP*-T7 | F: TAATACGACTCACTATAGGG  TACGGCGTGCAGTGCT  R: TAATACGACTCACTATAGGG  TGATCGCGCTTCTCG |  |
| pEDV6- *SmCSP4* | F: GGGGACAAGTTTGTACAAAAAAGCAGGCTTC  GCGCCGCAAAAAGATGC  R:GGGGACCACTTTGTACAAGAAAGCTGGGTC  TTAAAATTTAACAACCCCTT | Delivering by T3SS in wheat |
| pEDV6- *MpCSP4* | F: GGGGACAAGTTTGTACAAAAAAGCAGGCTTC  GCGCCGCAAAAAGATGCTG  R: GGGGACCACTTTGTACAAGAAAGCTGGGTC  TTAAAATTTGACAACACCTT |  |
| pEDV6- *ApCSP4* | F: GGGGACAAGTTTGTACAAAAAAGCAGGCTTC  GCGCCGCAAAAAGATGCTGC  R: GGGGACCACTTTGTACAAGAAAGCTGGGTC  TTAAAATTTAACAACACCTT |  |
| pEDV6- *AgCSP4* | F: GGGGACAAGTTTGTACAAAAAAGCAGGCTTC  GCGCCACAAAAAGATGCCGT  R: GGGGACCACTTTGTACAAGAAAGCTGGGTC  TTAAAATTTAGTAAAACCCTT |  |
| *FAD* | F: TCCCATTCCACCTACTGC  R: GGACTCACCAATCCGAGA | qPCR for wheat |
| *LOX* | F: GACCAGCGAAACAACAACC  R: GCATACAATAGCGGGAACAC |  |
| *PAL* | F: CCACCCTGGACAGATTGAA  R: ATGAGCGGGTTGTCGTTG |  |
| *PR1* | F: ATAACCTCGGCGTCTTCAT  R: TACTCGCTCGGTCCCTCT |  |
| *PR5* | F: CAAGCAGTGGTATCAACGCAGAG  R: GTGAAGCCACAGTTGTTCTTGATGTT |  |
| *DMR6* | F: GCTTCTTCCAGGTGCTCAAC  R: GGAGTAGAGCTTGGCCTTCT |  |
| *TaWRKY76* | F: GTCCTGCCGTGTCAAGAAGA  R: AACGCCCTCTGGAATTCTGG |  |
| *β-Actin* | F: GGAAAATCAGTCTCGGTTCAG  R: TCATACAGCAGGCAAGCAC |  |
| pGBKT7-*SmCSP4* | F:GGAATTCCATATGATGGCGCCGCAAAAAGATGCT  R: CGCGGATCCTTAAAATTTAACAACCCCTT | Y2H |
| T7 | F: TAATACGACTCACTATAGGG |  |
| 3’-BD | R: TAAGAGTCACTTTAAAATTTGTATAC |  |
| 3’-AD | R: AGATGGTGCACGATGCACAG |  |
| *SmCSP4*-*YFP* | F: GTGGATCCATGGCGCCGCAAAAAGATG  R: GGGAGCTCAAATTTAACAACCCCTTT | BiFC |
| *TaWRKY76*-*YFP* | F: GTGGATCCATGTGTTGCTTCTGGACCA  R: GGGAGCTCATAATCCGGCAGCTTCCG |  |
| *SmCSP4*-*MYC* | F: GTGGATCCATGGCGCCGCAAAAAGATG  R: GGGAGCTCAAATTTAACAACCCCTTT | CoIP |
| *TaWRKY76*-*HA* | F: GTGGATCCATGTGTTGCTTCTGGACCA  R: GGGAGCTCATAATCCGGCAGCTTCCG |  |
| *TaWRKY76* | F:TTTCTAAGGAAGGGCCAGAACCGGAAGCTGACTGAA  R:TTAACCACCACCACCGTGCAGACCTTCTTGACCTTG | VIGS |
| pGBKT7-*TaWRKY76* | GGAATTCCATATG ATGTGTTGCTTCTGGACC  CGCGGATCCCTAATAATCCGGCAGCTTC | Transcriptional activity |
| *proDMR6* | F: CCGGAATTCACTCCGGTAACGCTCCAATGATG  R: CGAGCTCGCCGGCACAAGAACAGAGAGAGA | Y1H |
| *proDMR6* | F: GGGGTACCACTCCGGTAACGCTCCAATGATG  R:CCCGAAGCTTGCCGGCACAAGAACAGAGAGAGA | Dual luciferase assay |
| *TaWRKT76* | F: GTGGATCCATGTGTTGCTTCTGGACC  R: GGACGTCCTAATAATCCGGCAGCTTC |  |
| *proDMR6* | F: GGGATCC ACTCCGGTAACGCTCCAATGATG  R: GCCCGGG GCCGGCACAAGAACAGAGAGAGA | GUS staining |
| *TaWRKY76* | F: GGGGTACC ATGTGTTGCTTCTGGACCAT  R: CCTCGAG CTAATAATCCGGCAGCTTC |  |
| *SmCSP4* | F: GGGGTACC ATGGCGCCGCAAAAAGATG  R: CCTCGAG AAATTTAACAACCCCTT |  |
